# Human-in-the-loop active learning for goal-oriented molecule generation

**DOI:** 10.1186/s13321-024-00924-y

**Published:** 2024-12-09

**Authors:** Yasmine Nahal, Janosch Menke, Julien Martinelli, Markus Heinonen, Mikhail Kabeshov, Jon Paul Janet, Eva Nittinger, Ola Engkvist, Samuel Kaski

**Affiliations:** 1 Department of Computer Science, Aalto University, 02150 Espoo, Finland; 2 Molecular AI, Discovery Sciences, R&D, AstraZeneca, 431 83 Mölndal, Sweden; 3 Department of Computer Science and Engineering, Chalmers University of Technology, 412 96 Gothenburg, Sweden; 4 Inserm Bordeaux Population Health, Vaccine Research Institute, Université de Bordeaux, Inria Bordeaux Sud-ouest, 33405 Talence, France; 5 Medicinal Chemistry, Research and Early Development, Respiratory and Immunology (R&I), R&D, AstraZeneca, 412 96 Gothenburg, Sweden; 6 Department of Computer Science, University of Manchester, Manchester, M13 9PL United Kingdom

**Keywords:** Goal-oriented molecule generation, Human-in-the-loop, Machine learning, Active learning, Interactive algorithms

## Abstract

**Abstract:**

Machine learning (ML) systems have enabled the modelling of quantitative structure–property relationships (QSPR) and structure-activity relationships (QSAR) using existing experimental data to predict target properties for new molecules. These property predictors hold significant potential in accelerating drug discovery by guiding generative artificial intelligence (AI) agents to explore desired chemical spaces. However, they often struggle to generalize due to the limited scope of the training data. When optimized by generative agents, this limitation can result in the generation of molecules with artificially high predicted probabilities of satisfying target properties, which subsequently fail experimental validation. To address this challenge, we propose an adaptive approach that integrates active learning (AL) and iterative feedback to refine property predictors, thereby improving the outcomes of their optimization by generative AI agents. Our method leverages the Expected Predictive Information Gain (EPIG) criterion to select additional molecules for evaluation by an oracle. This process aims to provide the greatest reduction in predictive uncertainty, enabling more accurate model evaluations of subsequently generated molecules. Recognizing the impracticality of immediate wet-lab or physics-based experiments due to time and logistical constraints, we propose leveraging human experts for their cost-effectiveness and domain knowledge to effectively augment property predictors, bridging gaps in the limited training data. Empirical evaluations through both simulated and real human-in-the-loop experiments demonstrate that our approach refines property predictors to better align with oracle assessments. Additionally, we observe improved accuracy of predicted properties as well as improved drug-likeness among the top-ranking generated molecules.

**Scientific contribution:**

We present an adaptable framework that integrates AL and human expertise to refine property predictors for goal-oriented molecule generation. This approach is robust to noise in human feedback and ensures that navigating chemical space with human-refined predictors leverages human insights to identify molecules that not only satisfy predicted property profiles but also score highly on oracle models. Additionally, it prioritizes practical characteristics such as drug-likeness, synthetic accessibility, and a favorable balance between exploring diverse chemical space and exploiting similarity to existing training data.

**Supplementary Information:**

The online version contains supplementary material available at 10.1186/s13321-024-00924-y.

## Introduction

Drug discovery is a notoriously time-consuming and expensive process. The effectiveness of exploring vast chemical spaces during the initial phases of the process is crucial, as it lays the groundwork for identifying promising candidate molecules [1]. In recent years, deep neural network architectures tailored to generative tasks have emerged as promising tools for accelerating early drug discovery, reducing time and financial investments [2–5].

While molecule generation can also be performed for exploratory purposes (i.e., non goal-oriented), for instance, to diversify chemical libraries for virtual screening [6], early drug discovery often involves generating molecules with specific properties such as drug-likeness or bioactivity [7–9]. The key to successful goal-oriented generation is to derive an accurate and representative scoring function that can evaluate molecules for the different desired properties, such that optimizing it results in generating desirable molecules. Once a suitable scoring function is established, the problem of generating desirable molecules can be framed as a discrete optimization problem, which can be effectively tackled using techniques such as reinforcement learning (RL) [10].

Quantitative Structure-Activity Relationship (QSAR) models predict the biological activity of chemical compounds based on their chemical structure. QSAR models represent a subset of broader quantitative structure–property relationship (QSPR) approaches, which encompass predictions of various molecular properties beyond bioactivity. These property predictors are commonly integrated into scoring functions to expedite the discovery of drug candidates and circumvent challenges in directly optimizing wet-lab measurements of target properties [11].

For instance, RL was employed to optimize a pre-trained Recurrent Neural Network (RNN) to generate binders for the Dopamine Receptor D2 (DRD2) based on QSAR predictions [10]. However, optimizing such predictors for molecule generation faces challenges as they often struggle to generalize post-deployment due to limited training data and evolving distributions during optimization [2]. Consequently, generative NNs guided by such predictors may yield sub-optimal molecules by overly relying on predictions in poorly understood chemical space regions [12, 13]. Our work addresses this particular challenge.

In the context of model deployment, methods have been proposed to monitor the generalization performance of property predictors when used for goal-oriented molecule generation [12–14]. A more continuous and dynamic approach to enhancing predictor generalization for this purpose involves leveraging active learning (AL).

AL is an experimental strategy that involves iterative selection of new data with the goal of minimizing the number of necessary training data while maximizing the gain in predictive accuracy and expanding the applicability domain (i.e., the range of chemical space where the model can make reliable predictions) [15]. An acquisition criterion is usually defined to select which experiments would contribute the most to an improved predictive accuracy. In the context of goal-oriented generation, AL can be used to encourage the generative agent to intentionally produce molecules that are poorly understood by the property predictor (e.g., by maximizing predictive uncertainty) which can then undergo experimental validation, serving as additional training data to enhance model generalization in subsequent generation cycles [2].

Typically, in current drug discovery pipelines, molecules that meet a specific target property profile (according to a target property predictor) are tested experimentally after each cycle [2]. However, immediate experimental labeling via wet-lab assays is often infeasible due to the significant time and monetary costs associated with synthesizing the compounds proposed by the generative model. Consequently, experimental labeling tends to be performed in batches rather than continuously. Moreover, previous studies have shown that the generated molecules with high predicted probability of meeting the target property profile often include many false positives according to wet-lab assays and *in-silico* oracles [2, 13]. At this stage, the property predictor needs to be refined (or replaced by alternative scoring functions), potentially involving manual intervention from human experts to acquire additional experimental data for training.

Human-in-the-loop (HITL) approaches were recently proposed to enhance the molecule generation process by allowing human experts to interact and provide feedback. For instance, these approaches enable the adaptation of scoring functions through RL with human feedback, ensuring that the generated molecules align better with desired properties [16, 17]. Based on empirical results showing the efficacy of a reward model trained on feedback from a chemistry expert regarding the optimization of DRD2 bioactivity [16], we posit that integrating feedback from well-aligned experts is crucial for ensuring that QSAR predicted scores which are optimized during the molecular generation process align well with the *true* scores of the target property.

In this regard, we propose to involve human experts in the AL process, allowing them to re-evaluate (approve or refute) predicted scores for newly generated molecules and incorporate them as additional training data to refine the target property predictor. We apply the Expected Predictive Information Gain (EPIG) acquisition strategy [18] which allows for prediction-oriented improvement, that is, favoring the acquisition of the most informative molecules to feed back to the property predictor to improve its predictive accuracy within specific regions of the chemical space (e.g., top-*N* ranked molecules). We empirically demonstrate the consistency of our HITL-AL approach in improving predictor’s generalization with respect to the *true* target function even under noisy and uncertain expert feedback. This was shown first through simulations using noisy oracles as experts, then through interacting with human experts in chemistry using the Metis user interface [19], highlighting the value of domain expertise as a proxy for experimental labelling in AL settings.

In summary, the contributions of our work are as follows:

- We leverage prediction-oriented data acquisition during goal-oriented generation processes to identify informative molecules for property predictors for which high predicted scores may not correspond to actual experimental outcomes.
- We offer chemistry experts the ability to confirm or refute property predictions and specify their confidence level, allowing for cautious predictor refinement.
- We demonstrate the practical application of our method through experiments involving different chemists, underscoring the importance of formulating precise questions for effective intervention.

**Fig. 1:**
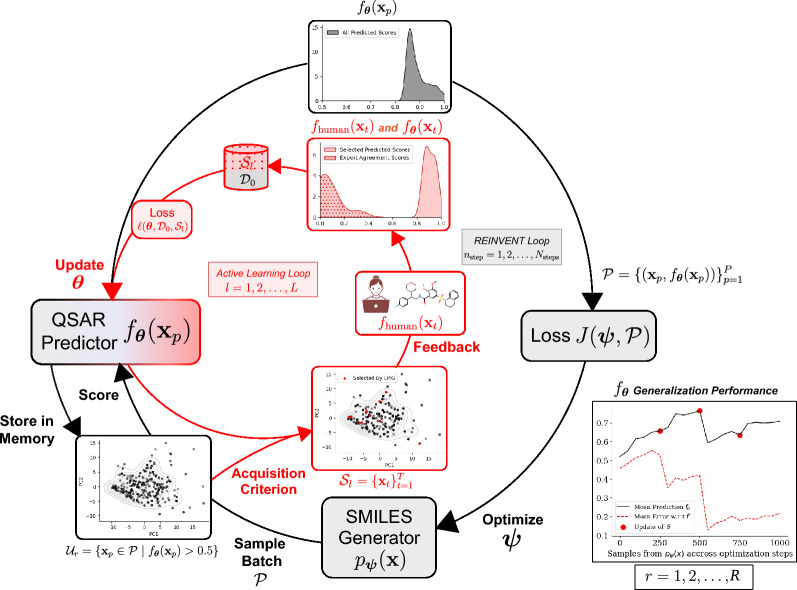
HITL-AL for goal oriented molecule generation. The method consists of two interleaved loops. The REINVENT loop (shown in black) describes the SMILES molecule generator whose parameters $\varvec{\psi }$ are optimized via stochastic gradient descent for a given number of steps $N_{\operatorname {steps}}$ to maximize the likelihood of generating high-scoring molecules by a target property predictor $f_{\varvec{\theta }}$. The Active Learning loop (shown in red), starting after the last step of each REINVENT loop, applies a data acquisition criterion to the property predictor $f_{\varvec{\theta }}$ and a pool ${\mathcal {U}}_r$ of high-scoring molecules generated via $p_{\varvec{\psi }}$ to select a set of queries ${\mathcal {S}}_l=\{{\textbf{x}}_t\}_{t=1}^T$ for a human expert (or a model of a human expert $f_{\operatorname {human}}$) to evaluate. Expert agreement scores $h_t$ with respect to the property predictions are collected in the form of additional training data ${\mathcal {S}}_l=\{({\textbf{x}}_t, h_t)\}_{t=1}^T$ used to fine-tune the predictor parameters $\varvec{\theta }$. The entire process can be repeated for $r=1,2,\dots ,R$ rounds, where each round *r* starts by initializing the SMILES molecule generator with the optimized parameters $\varvec{\psi }$ and replacing the property predictor in the REINVENT loss *J* with the updated one from the previous round $r-1$. As an example, we show on the bottom right of the figure how $f_{\varvec{\theta }}$ generalization to new samples from $p_{\varvec{\psi }}$ is improving, using $N_{\operatorname {steps}} = 250$ and $R = 4$

## Methods

### Goal-oriented molecule generation

We focus on goal-oriented molecular generation, meaning the design of molecules that satisfy one or multiple desired chemical properties, where some are available analytically and others are estimated by QSAR or QSPR models derived from experimental or simulation data.

While some target chemical properties can be computed analytically through first-principles methods (e.g., molecular weight) or through empirical, rule-based approaches (e.g., drug-likeness), most desired properties need to be estimated by data-driven QSAR or QSPR models. This can be the case for many reasons: (1) some chemical properties, such as bioactivity, are too complex to be estimated solely from a fixed set of predefined rules; (2) machine learning (ML)predictions are much cheaper to obtain compared to resource-intensive wet-lab measurements or physics-based simulations; or (3) there is a sufficient amount of labelled data that can serve as a starting point for faster convergence to target chemical spaces. We denote properties modeled from data as $f_{\varvec{\theta }}$ to distinguish them from properties $\phi$ that can be computed analytically.

Goal-oriented generation is usually framed as a multi-objective optimization problem [20] where the aim is to maximize a scoring function defined as

1 $$\begin{aligned} s({\textbf{x}}) = \sum _{j=1}^{J} w_j \sigma _j\left( \phi _j({\textbf{x}})\right) + \sum _{k=1}^{K} w_k \sigma _k \left( f_{\varvec{\theta }_k} ({\textbf{x}})\right) \end{aligned}$$

where ${\textbf{x}} \in {\mathbb {R}}^D$ is a vector representation of a molecule, $\phi _j: {\mathbb {R}}^D \rightarrow {\mathbb {R}}$ and $f_{\varvec{\theta }_k}: {\mathbb {R}}^D \rightarrow {\mathbb {R}}$ are the evaluation functions for the $j^{th}$ and $k^{th}$ desired properties respectively, and $w_j$ and $w_k$ their contributions to the overall objective or score, with $w$ in $]0,+\infty )$. Transformation functions $\sigma$ can be used to map evaluation functions to [0, 1]. The choice of these transformations depends on the desired property value. For instance, if the desired property value lies within a specific range of values [*a*, *b*], then $\sigma$ could be a double sigmoid function (Appendix C) with low and high parameters set to *a* and *b* respectively. Finally, weights *w* are normalized resulting in an overall score within [0, 1], facilitating interpretation; a score closer to 1 indicates greater suitability with respect to the overall objective.

### Target property predictor

In this study, we simulate wet-lab experimental labeling and operate under the condition that ground truth values for a given target property $k$ are consistently provided by an oracle $f^\star ({\textbf{x}})$. In our simulated environment, we have direct access to these oracle values for evaluating our models, rather than assuming their existence. To streamline the molecule generation process, we optimize a proxy $f_{\varvec{\theta }}$ of the oracle $f^{\star }$ to discover novel molecules.

This proxy is typically derived through supervised learning from available data ${\mathcal {D}}_0 = \{({\textbf{x}}_i, y_i)\}_{i=1}^{N_0}$, where ${\textbf{x}}_i \in {\mathbb {R}}^D$ represents a vector of $D$-dimensional count fingerprints for molecule $i$, and $y_i$ denotes its corresponding target value provided by the oracle $f^\star ({\textbf{x}}_i)$. Specifically, $y_i = f^\star ({\textbf{x}}_i) \in {\mathbb {R}}$ for regression tasks, and for classification tasks, $$y_i = {\left\{ \begin{array}{ll} 1 & \text {if } f^\star ({\textbf{x}}_i)> \delta \\ 0 & \text {otherwise} \end{array}\right. }$$, where $\delta$ is a predefined threshold.

In this work, we employ random forest (RF) models [21] as property predictors for molecule generation. This choice stems from their demonstrated robustness to small perturbations in high-dimensional feature spaces compared to deep neural networks and linear regression models [22]. Moreover, RF models have been found to be hard to outperform by deep models on a variety of molecular property prediction tasks, thus they are a robust baseline for our problem [23].

When deployed for goal-oriented generation, RF predictions are computed as

2 $$\begin{aligned} f_{\varvec{\theta }}({\textbf{x}}) = \frac{1}{B} \sum _{i=1}^{B} f_{\theta _i}({\textbf{x}}), \end{aligned}$$

where $B$ denotes the number of decision trees in the forest, $\theta _i$ represents the learned parameters of the $i$-th tree, and $\varvec{\theta } = \{\theta _1, \dots , \theta _B\}$ encompasses all tree parameters. For regression tasks, $f_{\theta _{i}}({\textbf{x}}) \in {\mathbb {R}}$ denotes the predicted value by the $i$-th tree, while for classification tasks, $f_{\varvec{\theta }}({\textbf{x}}) \in [0, 1]$ signifies the predicted probability of the positive class, which is obtained by averaging the discrete predictions $f_{\theta _i}({\textbf{x}}) \in \{0, 1\}$ from individual trees in the ensemble. Further details regarding the training procedure of the RF model can be found in Appendix A.

### Optimizing the molecule generator (REINVENT Loop)

We perform molecule generation using REINVENT [10], an algorithm employing an RNN for SMILES sequence generation and policy-gradient RL to guide the generation towards new SMILES that exhibit the desired properties according to a flexible scoring function, $s({\textbf{x}})$ (Eq. 1), that can include various scoring components. In this work, we specifically address the case where $s({\textbf{x}})$ includes at least one QSAR or QSPR model as a scoring component (i.e., $K>0$).

The RNN weights, which we denote as $\varvec{\psi }$, are first initialized with those of a pre-trained RNN $\varvec{\psi }_0$ on ChEMBL data [24], then optimized to generate molecules ${\textbf{x}}$ that maximize the reward given by the scoring function $s({\textbf{x}})$. At each optimization step, the scoring function evaluates a batch ${\mathcal {P}}$ composed of *P* generated molecules, and the resulting reward is used to tune the RNN weights $\varvec{\psi }$. More precisely, this is done through stochastic gradient descent with respect to a loss function $J({\mathcal {P}})$ defined as

3 $$\begin{aligned} J({\mathcal {P}}) = \frac{1}{P} \sum _{p=1}^P \left[ \log p_{\varvec{\psi }_0}({\textbf{x}}_p) - \lambda s({\textbf{x}}_p) - \log p_{\varvec{\psi }}({\textbf{x}}_p)\right] ^2 \end{aligned}$$

that represents the agreement between the pre-trained RNN log-likelihood $\log p_{{\varvec{\psi }_0}}({\textbf{x}}_p)$ modulated by the reward $s({\textbf{x}})$ and the agent log-likelihood $\log p_{{\varvec{\psi }}}({\textbf{x}}_p)$ of the generated molecules in ${\mathcal {P}}$. The agent log-likelihood $\log p_{{\varvec{\psi }}}({\textbf{x}}_p)$ refers to the probability of generating a SMILES sequence ${\textbf{x}}_p$ token by token, computed as $\log p_{\varvec{\psi }}({\textbf{x}}_p) = \sum _{t=1}^T \log p(x_t \mid x_{t-1}, \ldots , x_{1})$, where each $x_t$ is a token in the sequence. The influence of $s({\textbf{x}})$ is controlled by the pre-defined hyperparameter $\lambda$.

Additionally, at each optimization step, REINVENT stores a set ${\mathcal {M}}_r$ of chemically valid and structurally diverse molecules that have scores above a pre-defined threshold value. This memory set is formed via the Diversity Filters functionality in REINVENT , which ensures that generated molecules are added to different scaffold-specific buckets if they meet the score threshold. These filters discourage the generation of similar molecules once a scaffold-specific bucket reaches capacity.

A generation cycle with REINVENT completes (i.e., returning the final set ${\mathcal {M}}_r$) once a pre-defined number of optimization steps is reached. In a classical setting (i.e., without active human intervention), $s({\textbf{x}})$ is assumed to be static throughout *R* generation cycles. In our setting, we describe how $s({\textbf{x}})$ is adapted at every generation cycle after fine-tuning the property predictor with human feedback.

This procedure is referred to as the “REINVENT Loop” in Fig. 1.

### Fine-tuning the target property predictor (Active Learning Loop)

We introduce an AL loop following each generation cycle where a data acquisition criterion is employed to select *L* batches of *T* predicted high-scoring molecules from a pool ${\mathcal {U}}_r$, aiming to enhance the property predictor $f_{\varvec{\theta }}$ towards a specified objective. For instance, the EPIG acquisition criterion selects molecules whose observation by the predictor could reduce its predictive uncertainty at given inputs of interest (Eq. 5). For $l=1,\dots ,L$, each selected batch ${\mathcal {S}}_l$ undergoes evaluation by a human expert to ensure relevance of high-scoring molecules with respect to the target property. To streamline human interaction and optimize sample efficiency, a small batch size *T* is utilized.

During evaluation, each molecular structure, denoted as ${\textbf{x}}_t$ within the batch is presented via an interactive GUI interface. In simulated scenarios, it is routed to a surrogate model of the human expert, denoted as $f_{\operatorname {human}}$. The expert assesses each structure and provides an agreement score $u_t = f_{\operatorname {human}}({\textbf{x}}_t) \in [0,1]$ quantifying the extent to which they agree with its association to a high predicted score. The label $h_t$ for the evaluated molecule is then derived from this score $u_t$.

Upon evaluating *T* molecules, the target property predictor undergoes fine-tuning by incorporating the newly acquired data points. Specifically, predictor parameters $\varvec{\theta }$ are updated to minimize prediction errors with respect to ground truth labels $y_i$ for initial training samples in ${\mathcal {D}}_0$ and expert-provided labels $h_t$ for all newly acquired samples in ${\mathcal {D}}_r = \{{\mathcal {D}}_{r-1} \cup {\mathcal {S}}_l\}$ as follows

4 $$\begin{aligned} \varvec{\theta } = \underset{\varvec{\theta }}{\text {argmin}~}~ \frac{1}{N_0} \sum _{i=1}^{N_0} \ell (f_{\varvec{\theta }}({\textbf{x}}_i), y_i) + \frac{1}{T} \sum _{t=1}^{T} u_t \ell (f_{\varvec{\theta }}({\textbf{x}}_t), h_t) \end{aligned}$$

where $u_t \in [0,1]$ are considered as the confidence scores assigned by the human expert which directly influence the weighting of each new observed sample during predictor retraining, with higher expert confidence scores exerting greater influence.

This sample weighting strategy acknowledges the uncertainty associated with expert assessments, particularly when agreement with model predictions is ambiguous (e.g., $u_t \approx 0.5$). Such inputs provided by the expert are deemed less reliable and thus have less impact during predictor retraining. Finally, the updated predictor replaces the previous one in the scoring function for subsequent generation cycles.

This procedure is referred to as the “Active Learning Loop” in Fig. 1.

Both loops can be repeated for a given number of iterations *R*.

#### Data acquisition criteria

At the completion of a generation cycle (i.e., $n_{\operatorname {step}} = N_{\operatorname {steps}}$), an acquisition criterion is used to select a molecule ${\textbf{x}}_t$ for the human expert to evaluate. In typical AL settings, molecules would be selected from a pool available before training the property predictor. In our setting, molecules are selected from a pool ${\mathcal {U}}_r = \{{\textbf{x}}_m\}_{m=1}^M$ which corresponds to a set of high-scoring molecules stored in memory ${\mathcal {M}}_r$ across all optimization steps from the completed generation cycle. The acquisition criterion depends on the property predictor $f_{\varvec{\theta }}$. In this study, we compare the performance of different acquisition criteria against a random sampling baseline:

- Expected Predictive Information Gain (EPIG) [18] measures how much learning about a given data point (molecule) can improve predictions across other unseen data points, drawn from a target input distribution $p_{\star }({\textbf{x}}_{\star })$.
  An intuitive way to understand EPIG is to think of it as estimating how much the predictive uncertainty for molecules of interest ${\textbf{x}}_{\star }$ (e.g., the top 1% of high-scoring molecules) will decrease after receiving human feedback on a specific molecule ${\textbf{x}}$ sampled from the generated pool ${\mathcal {U}}_r$. In other words, EPIG asks *“How useful will this molecule be in reducing uncertainty about future promising molecules?”*. The higher the EPIG score, the more likely it is that knowing the true evaluation of this molecule will improve the property predictor ability to recognize optimal molecules in the future.
  Mathematically, EPIG can be formulated as the expected mutual information between $y$ and $y_{\star }$ given ${\textbf{x}}$ and ${\textbf{x}}_{\star }$, which can be written as an expected KL divergence between the joint distribution $p(y, y_{\star } \mid {\textbf{x}}, {\textbf{x}}_{\star })$ and the product of marginals $p(y \mid {\textbf{x}})p(y_{\star } \mid {\textbf{x}}_{\star })$:
  5 $$\begin{aligned} \texttt {EPIG}({\textbf{x}}) = {\mathbb {E}}_{p_{\star }({\textbf{x}}_{\star })} \big [\operatorname {KL}[p(y, y_{\star } \mid {\textbf{x}}, {\textbf{x}}_{\star }) \mid \mid p(y \mid {\textbf{x}})p(y_{\star } \mid {\textbf{x}}_{\star })]\big ] \end{aligned}$$
  where $$p(y \mid {\textbf{x}}) = {\mathbb {E}}_{p(\varvec{\theta } \mid {\mathcal {D}}_r)}[p(y \mid {\textbf{x}}, \varvec{\theta })]$$, $$p(y_{\star } \mid {\textbf{x}}_{\star }) = {\mathbb {E}}_{p(\varvec{\theta } \mid {\mathcal {D}}_r)}[p(y_{\star } \mid {\textbf{x}}_{\star }, \varvec{\theta })]$$, and $$p(y, y_{\star } \mid {\textbf{x}}, {\textbf{x}}_{\star }) = \int p(y \mid {\textbf{x}}, \varvec{\theta }) p(y_{\star } \mid {\textbf{x}}_{\star }, \varvec{\theta }) p(\varvec{\theta } \mid {\mathcal {D}}_r) \, d\varvec{\theta }$$.
  Molecules associated with the *T* highest EPIG scores are selected to form a batch ${\mathcal {S}}_l$.
  As outlined in Bickford Smith et al. [18], the EPIG criterion requires defining a conditional predictive distribution $p(y \mid {\textbf{x}})$ for each ${\textbf{x}}\in {\mathcal {U}}_r$ and a target input distribution $p_{\star }({\textbf{x}}_{\star })$. In the context of RFs, each decision tree $f_{\theta _i}$ is treated as an individual parameter value $\theta _i$. Consequently, each prediction $f_{\theta _i}({\textbf{x}})$ can be interpreted as a result of conditioning on $\theta _i$. This yields a collection of predictions $\{f_{\theta _i}({\textbf{x}})\}_{i=1}^B$ conditioned on *B* parameter values. By averaging over *B*, we obtain a conditional predictive distribution $p(y \mid {\textbf{x}})$, where $y$ represents the target class label for RF classifiers or the target value for RF regressors.
  To apply EPIG for reducing uncertainty about future promising molecules, we define the target input distribution $p_{\star }({\textbf{x}}_{\star })$ as the probability density function of the distribution of top-*k* molecules associated with the highest predicted scores in the pool ${\mathcal {U}}_r$:
  6 $$\begin{aligned} p_{\star }({\textbf{x}}_{\star }) = {\left\{ \begin{array}{ll} \frac{\sigma \left( f_{\varvec{\theta }}({\textbf{x}}_{\star }) \right) }{\sum _{{\textbf{x}}\in {\mathcal {U}}_r^{\operatorname {top}k}} \sigma \left( f_{\varvec{\theta }}({\textbf{x}}) \right) } & \text {if} \quad {\textbf{x}}_{\star } \in {\mathcal {U}}_r \\ 0 & \text {otherwise} \end{array}\right. } \end{aligned}$$
  where $\sigma \left( f_{\varvec{\theta }}({\textbf{x}}_{\star }) \right) \rightarrow [0,1]$ represents the predicted score for molecule ${\textbf{x}}_{\star }$, and the denominator is the sum of predicted scores over all top-*k* molecules in the pool ${\mathcal {U}}_r$. In our experiments, we set the top number *k* to 1000.
- Greedy corresponds to the predicted score for each ${\textbf{x}}\in {\mathcal {U}}_r$.
  7 $$\begin{aligned} \texttt {Greedy}({\textbf{x}}) = \sigma \left( f_{{\varvec{\theta }}}({\textbf{x}}) \right) \end{aligned}$$
  where $$\sigma : f_{{\varvec{\theta }}}({\textbf{x}}) \rightarrow [0,1]$$.
  Molecules associated with the *T* highest predicted scores are selected.
- Uncertainty quantifies the predictor uncertainty for each ${\textbf{x}}\in {\mathcal {U}}_r$.
  Since we are using RF models, we compute uncertainty as the disagreement or variance within the predictions made by the individual trees if $f_{\varvec{\theta }}({\textbf{x}}) \rightarrow {\mathbb {R}}$
  8 $$\begin{aligned} \texttt {UncertaintyRegression}({\textbf{x}}_t) = \frac{1}{B} \sum _{i=1}^{B} \left( f_{{\theta }_i}({\textbf{x}}) - {\bar{f}}_{\varvec{\theta }}({\textbf{x}})\right) ^2, \end{aligned}$$
  or as the Shannon entropy [25] in the predicted probabilities if $f_{\varvec{\theta }}({\textbf{x}}) \rightarrow [0,1]$
  9 $$\begin{aligned} \texttt {UncertaintyClassification}({\textbf{x}}_t) = - \left[ f_{\varvec{\theta }}({\textbf{x}})\log f_{\varvec{\theta }}({\textbf{x}}) + (1-f_{\varvec{\theta }}({\textbf{x}}))\log (1-f_{\varvec{\theta }}({\textbf{x}}))\right] \end{aligned}$$
  Molecules associated with the *T* highest predictive uncertainties are selected.
  The key difference between uncertainty sampling and EPIG is that, while uncertainty quantifies the model’s confusion about a specific prediction (i.e., *“How unsure am I about this prediction?”*), EPIG looks at the potential information gained from learning about a data point to improve predictions on other inputs (i.e., *“How much will learning about this molecule reduce my overall uncertainty?”*). In summary, uncertainty focuses on a single point, while EPIG considers the broader impact of acquiring new information across the target dataset.
- Random. It is used to uniformly randomly sample *T* molecules from ${\mathcal {U}}_r$.

The full procedure is summarized in Algorithm 1.

**Algorithm 1:**
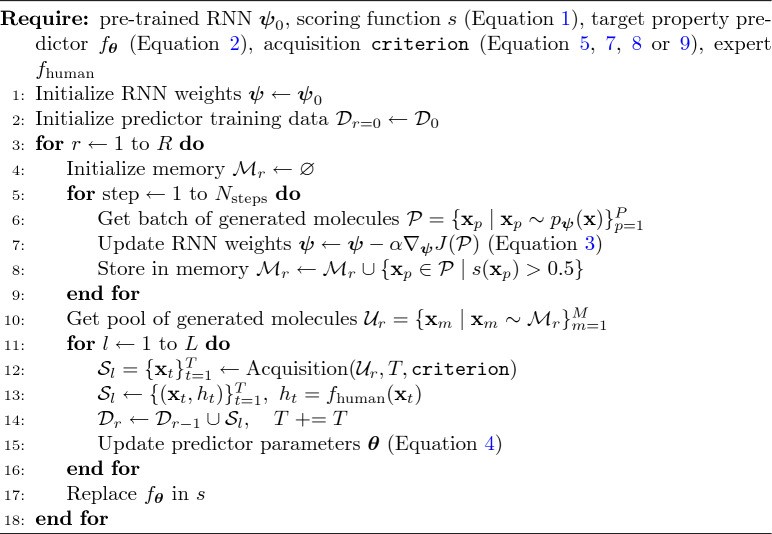
Fine-tuning the target property predictor $f_{\varvec{\theta }}$ for molecule generation

### Experiments

Our experiments aim to showcase the benefits of integrating human feedback through AL to refine target property predictors, compared to no predictor refinement (i.e., the predictor remains static throughout *R* rounds of the REINVENT loop). The improvement is measured in terms of error reduction between the predicted scores and the oracle scores associated with the generated molecules that the predictor identifies as promising for satisfying the target property. A diminishing error over *R* generation cycles indicates an enhanced predictor generalization to new molecules from REINVENT . This means that molecules identified as promising by the predictor are also promising according to the oracle. Consequently, optimizing a target property predictor that generalizes well outside its training domain results in generating more molecules that satisfy the target property.

We conducted various experiments, first involving simulated experts, then human experts providing feedback on molecules generated by REINVENT and optimized via RF predictors for specific target properties. Here, we outline two distinct use case scenarios: the first one focuses on optimizing molecules to achieve penalized LogP values within a defined desired interval, while the second targets the optimization of molecule bioactivity for a specific protein receptor, DRD2.

#### Use case 1: generation of molecules with optimal penalized LogP values

The aim of this use case is to generate new molecules with a penalized LogP within [2, 4], indicating sufficient lipophilicity for good absorption and distribution in the body. The penalized logP score of a molecule is defined as its octanol-water partition coefficient minus its synthetic accessibility (SA) score and number of long cycles [26].

We first train a penalized LogP predictor using a subet of 250 SMILES extracted from ChEMBL, then cleaned and filtered following Baltruschat and Czodrowski approach [27]. Extended Connectivity Morgan Fingerprints [28] of radius 3 (ECFP6) and length 2048 were generated using RDKit [29], alongside their associated LogP values which were calculated using the penalized LogP oracle described in Appendix D. We employed Scikit-learn [30] to fit a Random Forest Regressor (RFR) to all 250 ECFP6 vectors, optimizing hyperparameters (n_estimators=300 and min_samples_split=2) to minimize the Mean Squared Error (MSE) across 5 cross-validation folds. The resulting model achieved a Pearson linear correlation coefficient of 0.72 between true and predicted LogP values on a holdout test set of 600 SMILES from ChEMBL (Additional file 1: Figure S1).

For the REINVENT loop, we set the number of epochs $N_{\operatorname {steps}}$ to 250 and we keep the default value of 128 for batch size *P*. The hyperparameter $\lambda$ in the REINVENT loss function (Eq. 3) is also set to 128 by default. The scoring function comprises a single component which corresponds to the pre-trained RFR to which a double sigmoid transformation is applied to map the estimated LogP values to [0, 1], such that values lying within the desired range of [2, 4] result in transformed scores close to 1 (i.e., high reward scores). This double sigmoid transformation is illustrated in Appendix C.

For the Active Learning loop, we set the number of batches *L* to 5. We tried different query batch sizes $T=\{10,20,30\}$ and different data acquisition criteria (described in subsection 2.4.1) in separate trials. For each trial experiment, we chose to perform multiple iterations of batch AL instead of one, because of the advantages this may provide in terms of user experience and training efficiency. In fact, asking an expert to evaluate smaller batches iteratively instead of a larger batch at once may reduce the burden and prevent potential errors associated with fatigue and boredom from repetitiveness. Furthermore, iterative retraining on smaller batches can streamline the feedback process, since the expert can work with a model that becomes increasingly efficient at selecting the most informative or challenging instances in next AL iterations. Also, in cases where manipulation errors may occur (e.g., skipping a query or submitting an unintended answer), iterative batch active learning can help recover those. We set the number of rounds *R* to 4 in all trials.

To facilitate the execution of multiple trial experiments with various AL batch sizes and acquisition methods, we simulate an expert providing feedback by defining an expert surrogate as

10 $$\begin{aligned} f_{\operatorname {human}}({\textbf{x}}_t) = f^{\star }({\textbf{x}}_t) + \epsilon , \quad \epsilon \sim {\mathcal {N}}(0, \sigma _{\epsilon }) \end{aligned}$$

where $f^{\star }({\textbf{x}}) \in {\mathbb {R}}$ corresponds to the penalized LogP score oracle. This model assumes that, on average, an expert would accurately tell if a given query molecule ${\textbf{x}}_t$ is likely to exhibit moderate lipophilicity up to some normally-distributed noise. For each ${\textbf{x}}_t$, a noise value is sampled from a Gaussian distribution with mean 0 and standard deviation $\sigma _{\epsilon }$ to mimic cases where expert evaluation may deviate from that of the ground truth. We consider $\sigma _{\epsilon } = \{1.0, 5.0\}$ to evaluate our approach’s sensitivity to varying levels of noise in expert feedback. We also consider the ideal scenario where queries are directly evaluated by the oracle (i.e., $\epsilon = 0$).

#### Use case 2: generation of DRD2 binders

For this use case, we employed two distinct forms of feedback to refine the property predictor. The first, as described in Eq. 11, is an *in-silico* evaluator which assesses selected batches of molecules based on their predicted probabilities of DRD2 bioactivity. It was used to simulate expert feedback in our experiments. The second form of feedback involves real human assessments. Human experts evaluated the molecules through an interactive interface, providing agreement scores (ranging from 0 to 1) on proposed DRD2 actives, which were subsequently used to fine-tune the predictor.

**Mono-objective optimization.** For training the bioactivity predictor, we selected a subset of 240 SMILES from the ExCAPE database [31] such that they are representative of only two topological scaffolds. This was done to mimic usual real-world scenarios where existing experimental datasets are limited in size and diversity, especially in the early phases of drug discovery projects, and the aim is to explore the chemical space to identify novel molecules. ECFP6 vectors of length 2048 were generated using RDKit. Binary activity labels were obtained from a DRD2 oracle model described in Appendix D, resulting in 62 active samples and 178 inactive ones. After performing a 5-fold cross-validation, we fit a Scikit-learn Random Forest Classifier (RFC) with 200 estimators and a maximum tree depth of 10 to all 240 samples. Model performance was measured across different classification metrics described in Appendix A and reported in Additional file 1: Table S1.

We use the same settings as for the previous use case. The only difference is in the scoring function which comprises the pre-trained RFC predicting the probability of a given generated molecule to be active against DRD2. No transformation is applied in this case since predicted class probabilities always fall within the REINVENT score range of [0, 1].

We define the simulated expert for this use case as

11 $$\begin{aligned} f_{\operatorname {human}}({\textbf{x}}_t) = g\left( f^{\star }({\textbf{x}}_t) + \epsilon \right) \in [0,1], \quad \epsilon \sim {\mathcal {N}}(0, \sigma _{\epsilon }) \end{aligned}$$

where $f^{\star }({\textbf{x}}_t) \in [0,1]$ corresponds to the predicted positive class probability from the DRD2 oracle and $g: {\mathbb {R}}\rightarrow [0,1]$ is a clipping function ensuring that the transformed oracle score remains within the range [0, 1] after introducing the normally-distributed noise term $\epsilon$.

This approach assumes that, on average, an expert in DRD2 can generalize better to unseen molecules in the sense that their assessment would be better aligned with the $\textit{true}$ probability of DRD2 bioactivity. The noise term is added to the oracle score to simulate more realistic scenarios where an expert deviates or fails to perceive the $\textit{true}$ probability of DRD2 bioactivity for a given molecule. We consider $\sigma _{\epsilon } = \{0.15, 0.3\}$ as reasonably noisy experts, and $\sigma _{\epsilon } = \{0.5, 0.7\}$ as more extreme noise levels, as well as the ideal scenario where queries are directly evaluated using the oracle (i.e., $\epsilon = 0$).

**Multi-objective optimization.** This experiment describes more commonly encountered scenarios in drug discovery projects, where multiple objectives might be optimized simultaneously. We consider the task of generating molecules that are not only potential DRD2 binders but also tailored for high drug-likeness and minimal hERG bioactivity (i.e., minimizing the risk of hERG channel activation and subsequent arrhythmia [32]).

We then use $J=2$ additional objectives in the scoring function (Eq. 1). DRD2 and hERG bioactivity objectives are assigned equal weights ($w_0 = 1$ and $w_1 = 1$), while the drug-likeness objective, quantified via the Quantitative Estimation of Drug-likeness (QED) score, is assigned a weight of $w_2 = 0.5$.

The QED score is determined using the RDKit implementation [33], and the hERG bioactivity score is a probability value given by an oracle (described in Appendix D), to which a probability flipping transformation is applied, ensuring that molecules with lower hERG bioactivity probabilities get higher reward scores. The hERG bioactivity objective here was considered among the non-data-driven descriptors since our focus in this experiment is not to fine-tune it. We leave the fine-tuning of multi-task target property predictors for future work.

For the first iteration of the REINVENT loop, we set $N_{\operatorname {steps}}$ to 1200 so that the generator goes through a sufficient number of optimization steps to achieve a high balanced score between the three objectives, and therefore generate a representative initial pool ${\mathcal {U}}_r$ for the first iteration of the Active Learning loop. We set $\lambda$ to 180 in the REINVENT loss function to accelerate convergence to high reward scores from the multi-objective scoring function. $N_{\operatorname {steps}}$ was then reduced to 250 with the purpose of fine-tuning the generation process after observing expert feedback.

In the Active Learning loop, ${\mathcal {U}}_r$ is defined as the set of generated molecules with DRD2 bioactivity scores higher than 0.5. The simulated expert, as described in the mono-objective setting, provides a score representing their agreement level with the proposed molecules predicted as promising DRD2 binders.

#### Comparison with other approaches and different configurations

Considering the mono-objective generation setting, we evaluate how well our approach behaves compared to other strategies and configurations applied to the generator or the property predictor before or during the generation process. These configurations include:

- Initializing REINVENT with a generative agent pre-trained on the initial training set of the property predictor.
- Optimizing Tanimoto fingerprint similarity [34] between generated molecules and known molecules from the initial training set.
- Applying post-hoc calibration through the Platt Scaling method [35] to the property predictor before using it in REINVENT . Platt Scaling involves fitting a logistic regression model to a classifier’s raw scores, transforming them into calibrated probabilities using a sigmoid function.

Platt Scaling can only be applied to binary classification models, therefore we apply it to the DRD2 bioactivity use case only.

Moreover, we assess the impact of lower and higher human feedback frequency by setting the number of optimization steps in the REINVENT Loop $N_{\operatorname {steps}}$ to 150, 500 and 1000 in separate trials.

#### Human experiments

Finally, we validate our approach in a real-world, multi-objective molecule generation scenario where a chemistry expert interacts with the property predictor through the Metis GUI [19].

This experimental design mirrors our previous multi-objective generation setting focused on generating DRD2 actives with high QED and low hERG bioactivity. However, instead of relying on simulated expert feedback, we collect real feedback through the GUI that displays molecules selected through batch AL alongside their associated DRD2 predicted bioactivities.

**Fig. 2:**
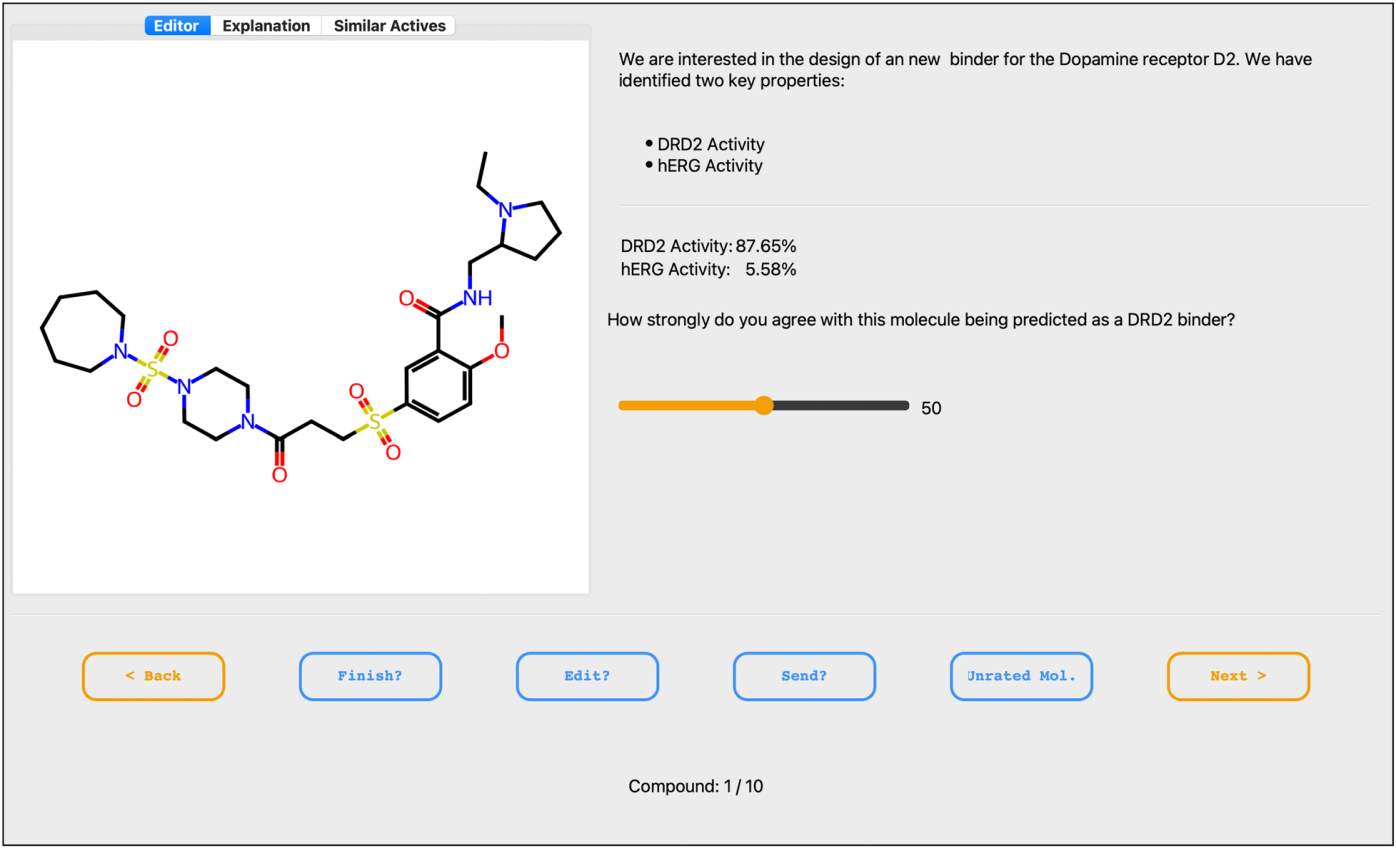
Metis GUI displaying molecules generated based on a DRD2 predictor, a hERG predictor and QED that were selected based on EPIG acquisition criterion for updating the DRD2 predictor

As shown in Fig. 2, the queried expert can answer how strongly they agree with a given selected molecule being predicted as DRD2 active using a slider ranging from 0 (strongly disagree) to $100\%$ (strongly agree). The slider value initializes at $50\%$, allowing the expert to maintain this value if they have no specific opinion about a given molecule. For instance, if the slider is positioned at $40\%$ by the expert, then the label $h_t$ given to the queried molecule is 0 since the slider value is lower than the threshold of $50\%$, while the confidence score $u_t = 100-40 = 60\%$, reflecting how much the expert was confident in their disagreement with the QSAR model prediction.

For each experiment, we started with the initial generative agent optimized in the previous experiment for 1200 steps then performed a total of 3 rounds, with $L=5$ batch AL iterations, using EPIG as acquisition criterion and a batch size *T* of 10 molecules.

We used Metis features to help the expert in providing more informed assessments about the selected molecules. These features include visual explanations of DRD2 bioactivity predictions, highlighting molecular fragments based on their positive and negative atomic contributions to bioactivity (Additional file 1: Figure S2), and a similarity search algorithm based on MACCS keys that selects the most similar active molecules from the initial training set of the DRD2 predictor (Additional file 1: Figure S3). All GUI settings used for these experiments, including which additional features were displayed on the Metis interface to support chemist feedback, are provided in Additional file 1: Listing S1.

In the subsequent section, we present results derived from three distinct human experiments, each involving a different expert in fine-tuning a DRD2 bioactivity predictor within the multi-objective generation scenario described above. The two first experts are experienced in generative chemistry and interact regularly with synthetic chemists to suggest useful solutions and adapt the generative tools to their needs. The third expert is more experienced in medicinal chemistry. All are co-authors of the manuscript.

## Results and discussion

### Simulated experiments

#### Use case 1: generation of molecules with optimal penalized LogP values

**Fig. 3:**
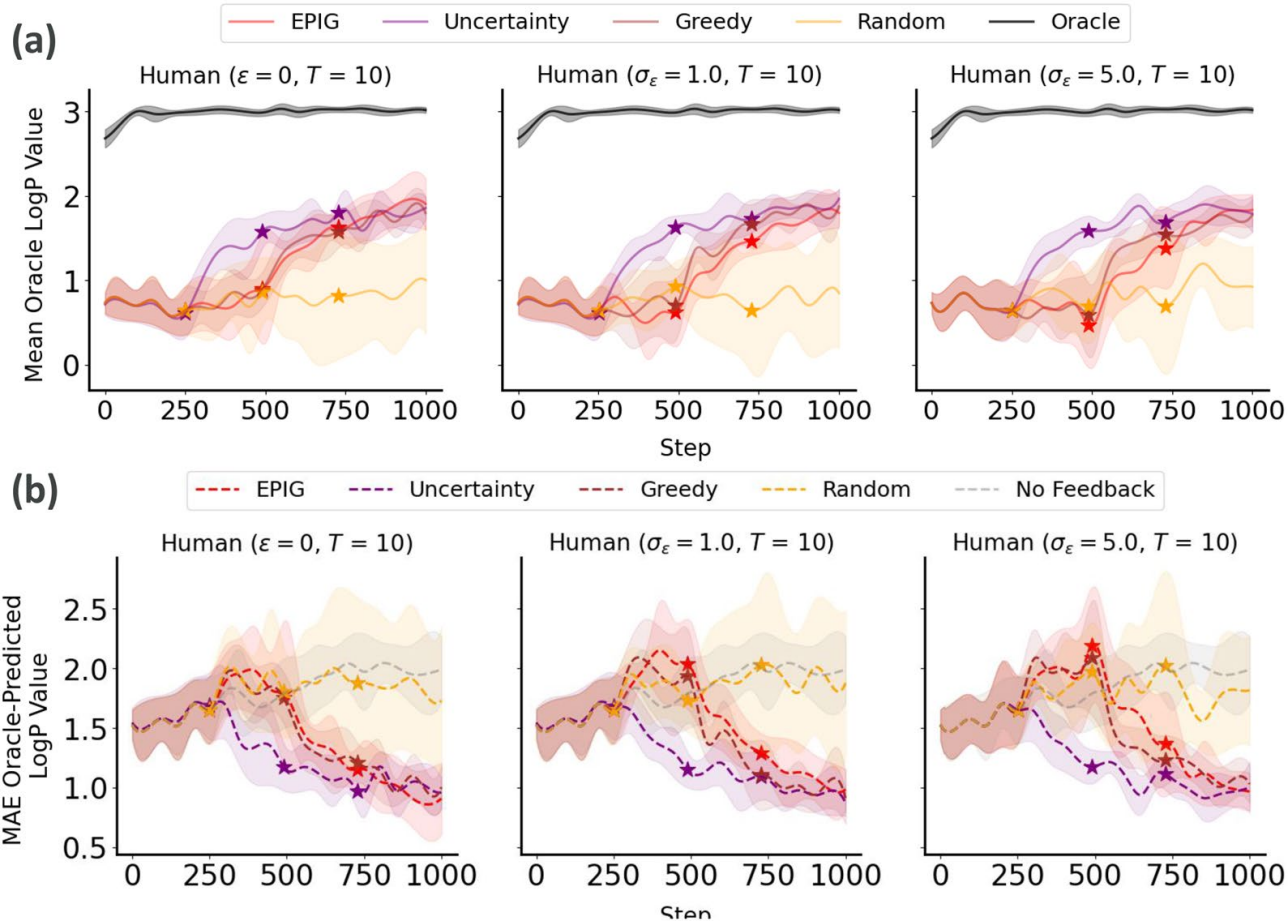
Generalization performance of the penalized LogP predictor to molecules generated at each step of the process. **a** Mean oracle score of molecules generated by optimizing the oracle itself (i.e., best-case scenario) and the fine-tuned LogP predictor. **b** MAE between predicted and oracle LogP values. For both metrics, we report the means and standard deviations across 10 different replicates of each experimental run. The start of the Active Learning loop at each round is illustrated by a star. The noise in simulated expert feedback increases from left to right

**Fig. 4:**
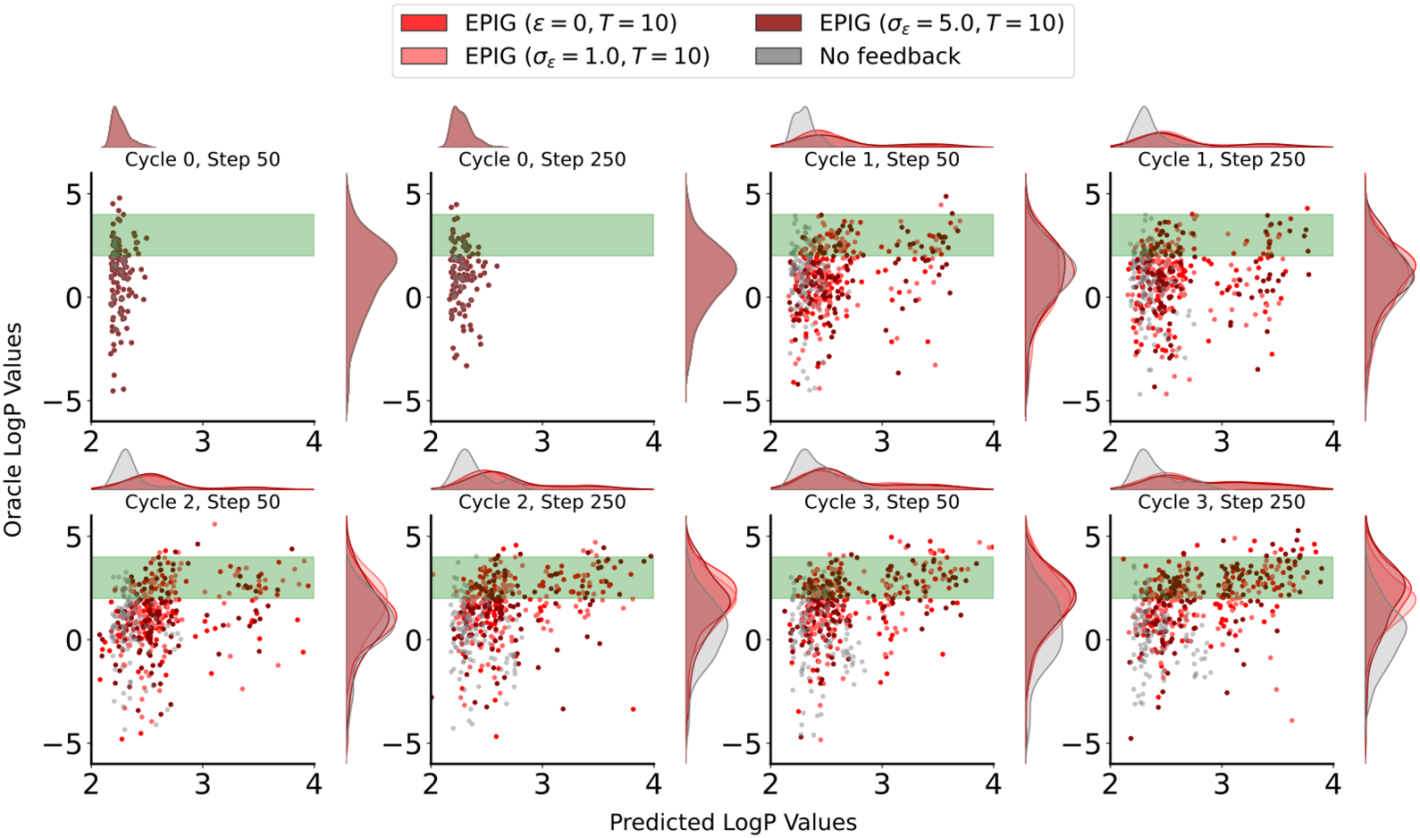
EPIG-guided active learning improves the generation of molecules with desired penalized LogP values according to the oracle compared to the “No Feedback” baseline. This improvement is visible in the concentration of points within the green rectangular target area and the increasing linear correlation between oracle and predicted LogP values. The plot shows the predicted LogP values better aligning with the oracle values over the course of the generation runs. We report these values for all generated molecules with a transformed predicted LogP score above 0.5. The generation runs with AL updates using EPIG as the acquisition criterion and noisy simulated experts are shown in red, while the “No Feedback” baseline is shown in gray

We initiated our investigation by assessing the efficacy of our approach under various acquisition strategies-random, uncertainty, greedy, and EPIG-while querying a simulated expert. This expert’s reliability was modeled with three noise levels: noise-free ($\epsilon = 0$), moderate noise ($\sigma _{\epsilon } = 1.0$) and high noise ($\sigma _{\epsilon } = 5.0$). Our findings indicate that integrating AL to update the penalized LogP predictor contributes to the progressive alignment of penalized LogP estimates with oracle values over time (i.e., reduction of the MAE, figure 3), resulting in the generation of molecules that are more likely to achieve the desired target (in this case, a penalized LogP within 2 and 4) according to the oracle (Fig. 4). Notably, these positive outcomes persist even in the presence of increased levels of noise in the simulated expert feedback, underscoring the robustness of our AL approach to unreliable inputs that may occur in real-world scenarios.

Our comparison of the four acquisition strategies revealed similar performance for EPIG, uncertainty and greedy sampling in improving the generalizability of the LogP predictor in terms of significantly reducing the MAE between estimated and oracle values, with greater performance than a random sampling baseline and a slightly higher performance when using model uncertainty as a criterion for data acquisition, followed by EPIG. To statistically validate these findings, we conducted an ANOVA test, which indicated significant differences across the acquisition strategies (F-statistic = 16.63, *p*-value $< 1 \times 10^{-10}$). Further pairwise comparisons using Tukey HSD test revealed several significant differences between the strategies. Specifically, EPIG, uncertainty, and greedy sampling methods significantly outperformed the “No Feedback” baseline in terms of mean LogP predictor accuracy, with *p*-values $< 5 \times 10^{-4}$. EPIG also significantly improved performance over random sampling (*p*-value = $2 \times 10^{-4}$), while no significant differences were observed between EPIG, uncertainty, and greedy sampling themselves. These statistical analyses confirm that the choice of acquisition strategy has a substantial impact on the improvement of the model accuracy, with EPIG and model uncertainty criteria showing the most promise.

Additional performance metrics, provided by the benchmarking platform MOSES [36] and described in Appendix E, for the final selection (i.e., resulting from the final optimization step) of high-scoring molecules stored in memory are summarized in Table 2. We can observe that expert inputs do not significantly deteriorate the internal diversity of final molecular sets in comparison with the “No Feedback” baseline. Our results also demonstrate that both EPIG and random sampling significantly improve the exploitation-exploration trade-off, which can be observed by a maintained internal diversity score and an increased similarity between the generated molecules and those present in the initial training set when comparing with the “No Feedback” baseline. Moreover, based on the similarity metrics measured between the sets of generated molecules and the expert queries that were selected using each acquisition criterion, EPIG appears to identify the additional training molecules which are the most informative for improving the generalizability of predictions for the current most promising designs. Moreover, using AL with expert feedback to refine the LogP predictor leads to the generation of more synthetically accessible molecules in comparison with the “No Feedback” baseline and the random sampling strategy.

##### Comparison with other approaches and different configurations

**Comparison with other approaches to improve model predictions.** Our approach was rigorously compared with several baselines to assess its effectiveness. One baseline involved constraining the molecule generator to remain close to the training set, ensuring generated molecules were similar to known examples. Another baseline used transfer learning to pre-train the molecule generator using the initial training set of the predictor, enhancing the generator’s focus on relevant chemical space from the start of the generation process. Among these approaches, constraining the generator with respect to training set similarity led to the lowest MAE between predicted and oracle values (Additional file 1: Figure S6). While this strategy resulted in lower MAEs, it imposed strong limitations on the type of chemistry that could be explored. Moreover, pre-training the molecule generator significantly improved the LogP predictor generalizability in comparison with the “No Feedback” baseline, as well as AL using EPIG, resulting in a lower MAE at the start of the molecule generation process, which increased as the process continued. Notably, when using EPIG for AL, the MAE progressively reduced, eventually matching the MAE achieved by the pre-trained generator.

**Impact of the number of human queries.** The number of human queries significantly impacts AL results, particularly when employing random sampling. With a lower number of selected queries, the performance of random sampling was suboptimal. As the number of human queries increased, the AL results using random sampling improved, highlighting the need of some acquisition strategies for larger query budgets. Even when using the lowest query budget $T=10$, the AL approach results in improved predictor generalization performance over time (Additional file 1: Figure S4).

**Optimal frequency of human queries.** The frequency of querying humans for feedback is another crucial factor influencing the performance of our AL approach. We tested different human querying frequencies (every 150, 250, 500 and 1000 generator optimization steps). Results are illustrated in Additional file 1: Figure S5. For updating regression models such as the LogP predictor, querying every 150 steps was found to be suboptimal, suggesting that the generator might not have adequate time to adapt from the feedback and converge to more optimal chemical spaces. Conversely, querying every 500 or 1000 steps may result in increasing the MAE between oracle and predicted values, indicating that less frequent updates can cause the predictor to not generalize well anymore to new explored regions of the chemical space. Through experimentation, querying every 250 steps emerged as the most optimal frequency. This balanced approach provided the model with sufficient time to integrate feedback and update its predictions effectively, ensuring better alignment with oracle values and improved overall generalization.

#### Use case 2: generation of DRD2 binders

##### Mono-objective setting

**Fig. 5:**
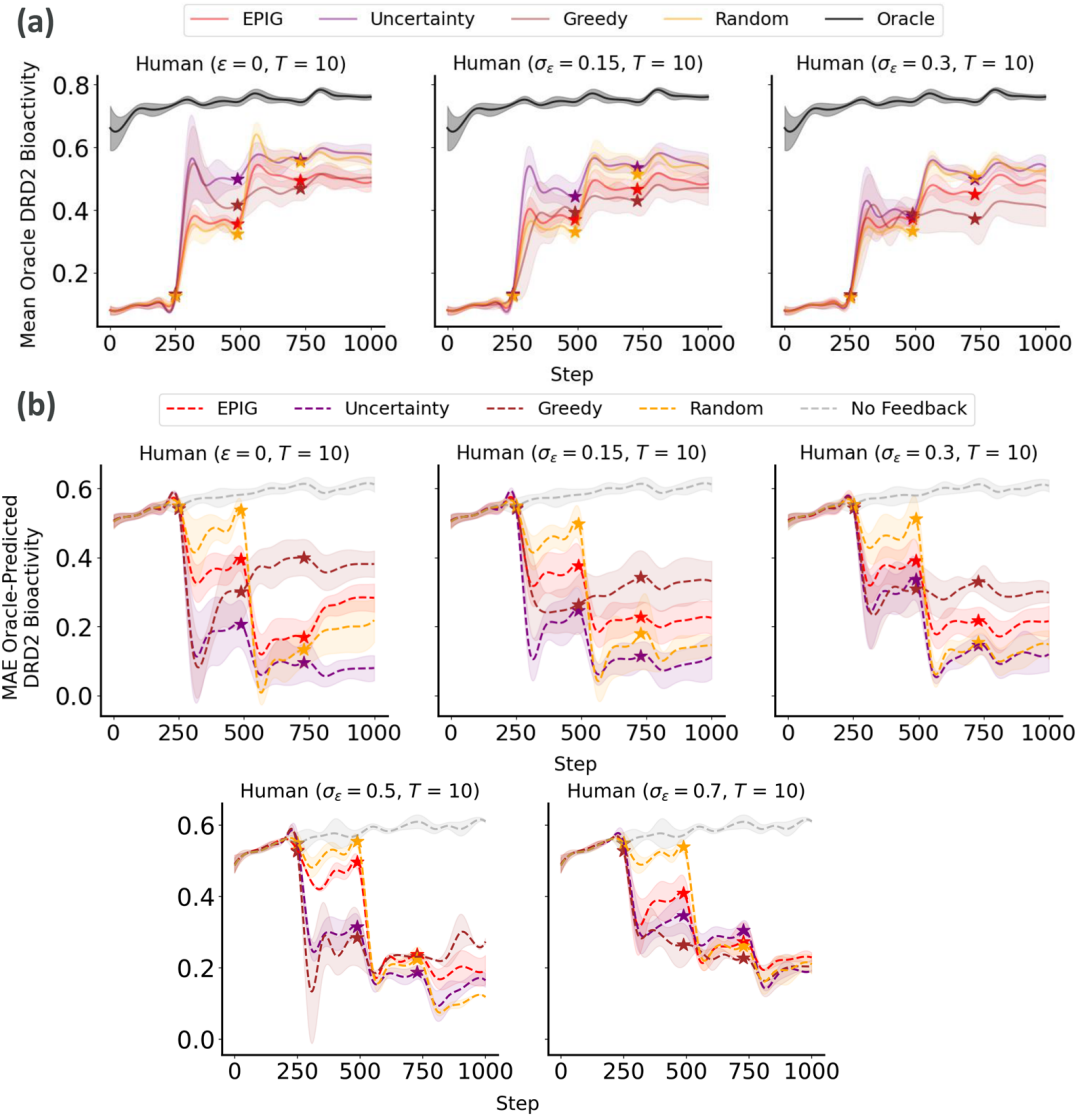
Generalization performance of the DRD2 bioactivity predictor to molecules generated at each step of the process. **a** Mean oracle score of molecules generated by optimizing the oracle itself (i.e., best-case scenario) and the fine-tuned DRD2 bioactivity predictor. **b** MAE between predicted and oracle DRD2 bioactivity scores. For both metrics, we report the means and standard deviations across 10 different replicates of each experimental run. The start of the Active Learning loop at each round is illustrated by a star. The noise in simulated expert feedback increases from left to right

We follow the same procedure to investigate the results from the generation of DRD2 bioactive molecules based on the DRD2 bioactivity predictor. We observe that, in comparison with the “No Feedback” baseline, all acquisition strategies resulted in improved alignment between predicted and oracle DRD2 values (measured in terms of MAE between predicted and oracle probabilities of being DRD2 active) even under the presence of increasingly noisy feedback (Fig. 5).

Among these strategies, uncertainty sampling emerges as the best performing one in terms of MAE reduction, outperforming EPIG, greedy, and random sampling strategies. Statistical analysis using the Tukey HSD test indicated significant differences between the “No Feedback” baseline and all acquisition strategies (ANOVA, $F(3, 36) = 19.87, \, p\text {-value} < 1.85 \times 10^{-12}$). Specifically, all acquisition methods significantly improved MAE compared to the baseline ($p\text {-value} < 0.001$), with uncertainty sampling showing the most substantial improvement. However, no significant difference was observed between uncertainty sampling and EPIG (mean difference = $-$0.1094, $p\text {-value} = 0.0723$), suggesting similar effectiveness in enhancing the DRD2 bioactivity predictor’s generalizability. The comparison between EPIG and greedy sampling yielded a mean difference of 0.0524 with a $p$-value of $0.717$, indicating no statistically significant difference. Similarly, the comparison between EPIG and random sampling methods resulted in a mean difference of $-$0.0062 with a $p$-value of $0.9999$, indicating no statistically significant difference.

The observed rise in the MAE between AL steps can be attributed to the fact that, after each update, the property predictor is again deployed to explore new regions of the chemical space, where prediction errors may become more likely. Following each AL step, the predictor receives expert feedback in the form of new training data points, which expands its knowledge of the current chemical space and reduces prediction errors (as reflected by the sharp dips in MAE). However, as the process progresses through subsequent REINVENT steps, the predictor increasingly ventures outside of its training distribution, leading to a gradual rise in MAE.

Additionally, the molecules generated under EPIG score highly for QED and are more synthetically accessible, as evidenced by a lower SA score compared to other acquisition strategies (Table 3). The underlying mechanism for why EPIG might result in molecules with higher synthetic accessibility and drug-likeness could be linked to both the nature of EPIG’s sampling strategy and the feedback given to the selected molecules via EPIG.

On the one hand, since EPIG aims to reduce predictive uncertainty in the most promising molecules, it might be inherently biased towards regions of the chemical space that are well-understood and well-represented in the training data. These regions are likely to contain molecules that are not only bioactive but also synthetically accessible and drug-like. On the other hand, the feedback provided by the noisy oracles on the selected molecules might implicitly consider factors like synthetic accessibility and drug-likeness in their evaluations. This feedback loop could gradually steer the model towards favoring molecules that score well on these practical metrics, even if they are not directly part of the initial scoring function. This could stem from the fact that the updated QSAR model might now use features related to drug-likeness and synthetic accessibility to predict bioactivity. Thus, when EPIG selects molecules to reduce uncertainty in bioactivity predictions, it might inadvertently select molecules with favorable synthetic accessibility and drug-likeness due to these underlying correlations.

**Fig. 6:**
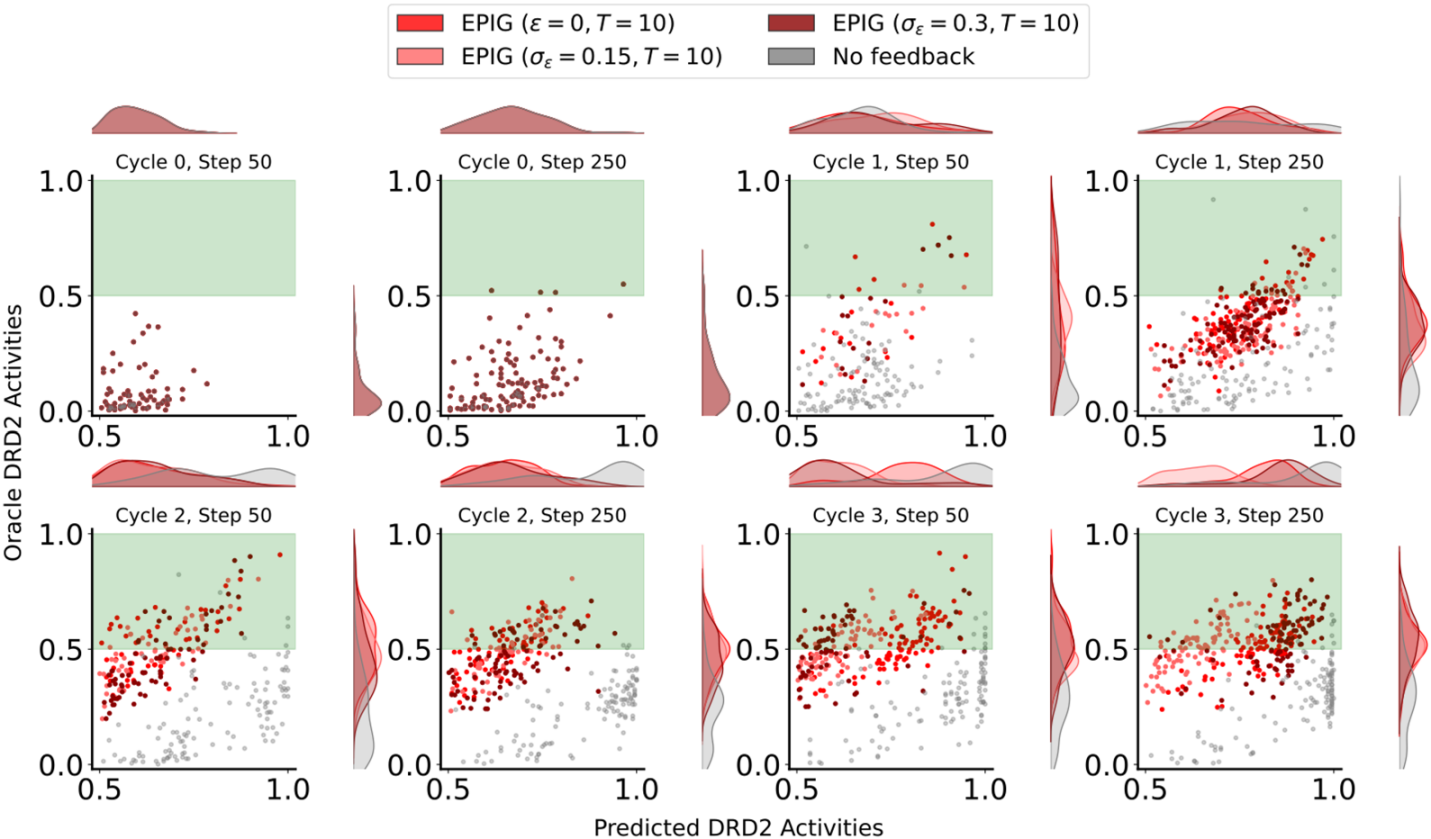
EPIG-guided active learning improves the generation of molecules with high probabilities of DRD2 bioactivity according to the oracle compared to the “No Feedback” baseline. We report the oracle vs predicted activity probabilities for all generated molecules with a predicted activity above 0.5. The generation runs where AL updates using simulated expert models with different noise levels were used are shown in red, while the “No Feedback” baseline is shown in gray

Despite uncertainty sampling resulting in higher performance on the MAE metric, EPIG’s advantages in producing more drug-like and synthetically feasible molecules make it a compelling choice. These factors are crucial for real human-in-the-loop experiments, where the goal is not only to predict DRD2 bioactivity accurately but also to discover drug-like candidates which are easy to synthesize. EPIG also appears robust to increased noise when simulating expert feedback, allowing for more efficient search for molecules with higher probabilities of being DRD2 active (Fig. 6). Therefore, we choose EPIG as the acquisition function for human experiments to improve the generalizability of the DRD2 predictor, leveraging its balanced performance across multiple critical metrics.

##### Multi-objective setting

**Fig. 7:**
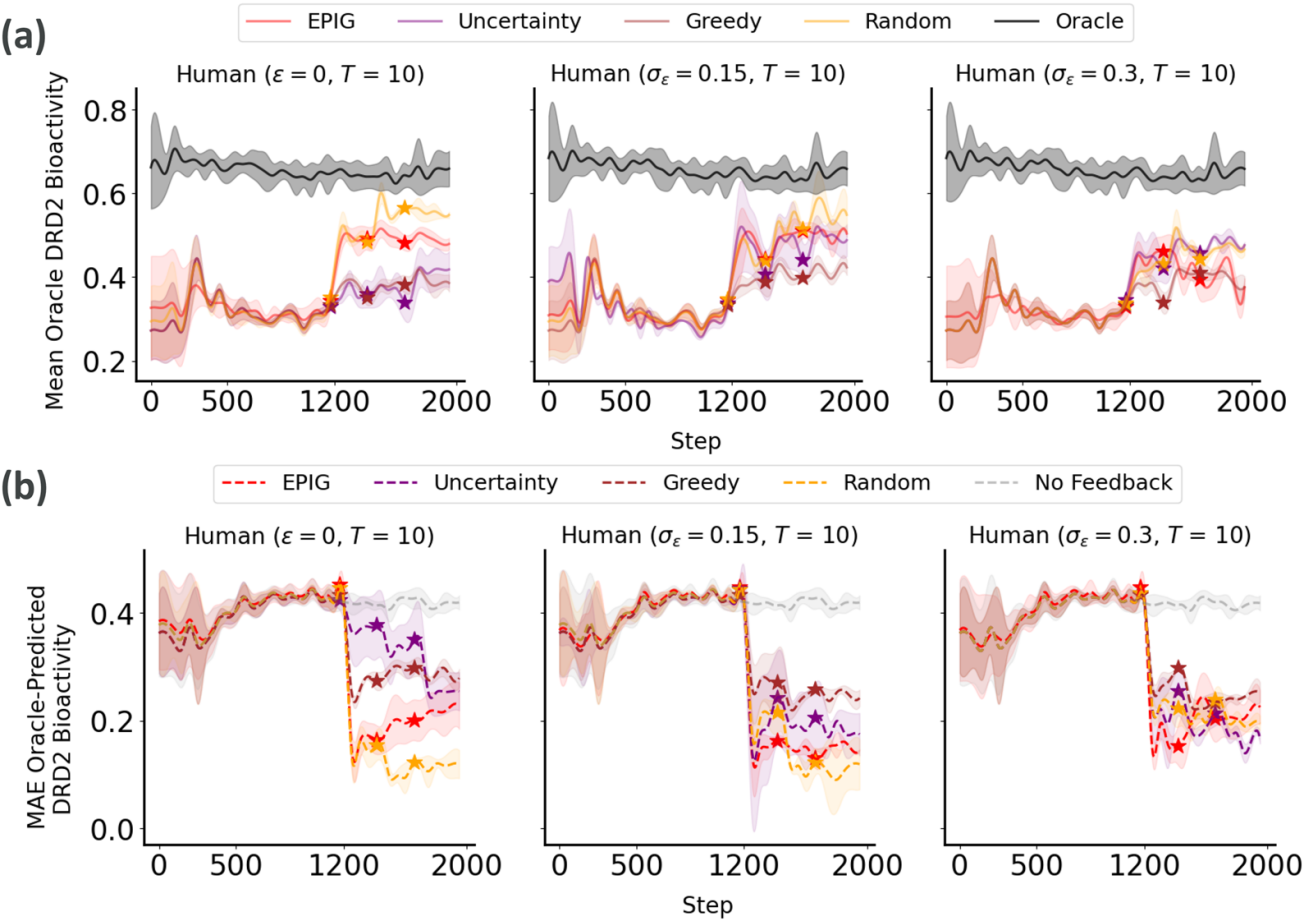
Generalization performance of the DRD2 bioactivity predictor to molecules generated at each step of the process (using a multi-objective scoring function). **a** Mean oracle score of molecules generated by optimizing the oracle itself (i.e., best-case scenario) and the fine-tuned DRD2 bioactivity predictor. **b** MAE between predicted and oracle DRD2 bioactivity scores. For both metrics, we report the means and standard deviations across 10 different replicates of each experimental run. The start of the Active Learning loop at each round is illustrated by a star. The noise in simulated expert feedback increases from left to right

In our exploration of multi-objective molecule generation, particularly targeting DRD2 bioactives with inactivity against hERG and high drug-likeness (QED), our focus was still on updating the DRD2 bioactivity predictor through AL. In this multi-objective setting, EPIG and random sampling under highly noisy feedback emerged as the most effective strategies in terms of improving the generalizability of the DRD2 bioactivity predictor and aligning its outputs with the oracle values, showcasing greater robustness and reliability (Fig. 7). ANOVA analysis indicated significant differences among acquisition strategies concerning their effectiveness in reducing the MAE for predicting DRD2 bioactivity ($F(3, 36) = 13.49$, *p*-value $8.40 \times 10^{-10}$). Post-hoc Tukey’s HSD tests revealed that EPIG (mean difference = $-$0.1101, *p*-value $< 0.001$), Greedy (mean difference = $-$0.0656, *p*-value = 0.008), Random (mean difference = $-$0.1311, *p*-value $< 0.001$), and Uncertainty sampling (mean difference = $-$0.0642, *p*-value = 0.010) significantly improved MAE compared to the baseline. Among pairwise comparisons between acquisition strategies, only Greedy vs. Random (mean difference = $-$0.0655, *p*-value = 0.008) and Random vs. Uncertainty (mean difference = 0.0669, *p*-value = 0.006) showed statistically significant differences. No significant differences were observed between EPIG and Greedy (*p*-value = 0.154), EPIG and Random (*p*-value = 0.815), EPIG and Uncertainty (*p*-value = 0.132), or Greedy and Uncertainty (*p*-value = 1.0).

However, the improvement in terms of drug-likeness (QED) and synthetic accessibility is less notable and not statistically significant in comparison with the “No Feedback” baseline, at the exception of the greedy sampling strategy which results in significantly higher mean QED score (Table 4). Maintaining a sufficient level of internal diversity as well as drug-likeness and synthetic accessibility within generated molecules is crucial in multi-objective optimization settings where the balance between generating DRD2 actives and useful molecules simultaneously is essential. EPIG also showed potential in balancing exploration (i.e., diversity of generated molecules) and exploitation (i.e., similarity between generated and training molecules), as well as a good ability to retrieve the most informative additional training molecules to update the model, which is evidenced by the similarity metrics between generated molecules and the selected queries to be evaluated by the expert. Based on the simulated experiment results, we use EPIG as the data acquisition method of choice for the following human experiments.

##### Comparison with other approaches and different configurations

**Comparison with other approaches to improve model predictions.** For the DRD2 bioactivity use case, we compare our approach against the same baselines. Additionally, we include a third baseline which corresponds to the generative agent optimizing the DRD2 bioactivity predictor calibrated beforehand using the Platt Scaling method. Our approach outperformed all three baselines in terms of reducing the MAE between predicted and oracle DRD2 activities for the generated molecules (Additional file 1: Figure S9).

**Impact of number of human queries.** Similar to the LogP use case, all data acquisition strategies result in improved predictor generalization performance over time even when using the lowest query budget $T=10$ (Additional file 1: Figure S7).

**Optimal frequency of human queries.** A similar generalization performance (i.e., reduction of MAE between predicted and oracle DRD2 bioactivity probabilities) was observed when querying the expert at different frequencies during the molecule generation process (every 150, 250, 500 and 1000 generator optimization steps). Results are illustrated in Additional file 1: Figure S8.

### Human experiments

Finally, we present the results derived from engaging with three distinct chemists, who were tasked with indicating their level of agreement, expressed through an interactive slider, regarding the potential DRD2 bioactivity of a given generated molecule. Each chemist was requested to provide this assessment for a total of 50 molecules during each interaction round. To enhance chemist decision-making, we additionally provided them with images depicting the most similar active molecules to each newly generated molecule that are already present in the initial training set of the DRD2 predictor.

**Fig. 8:**
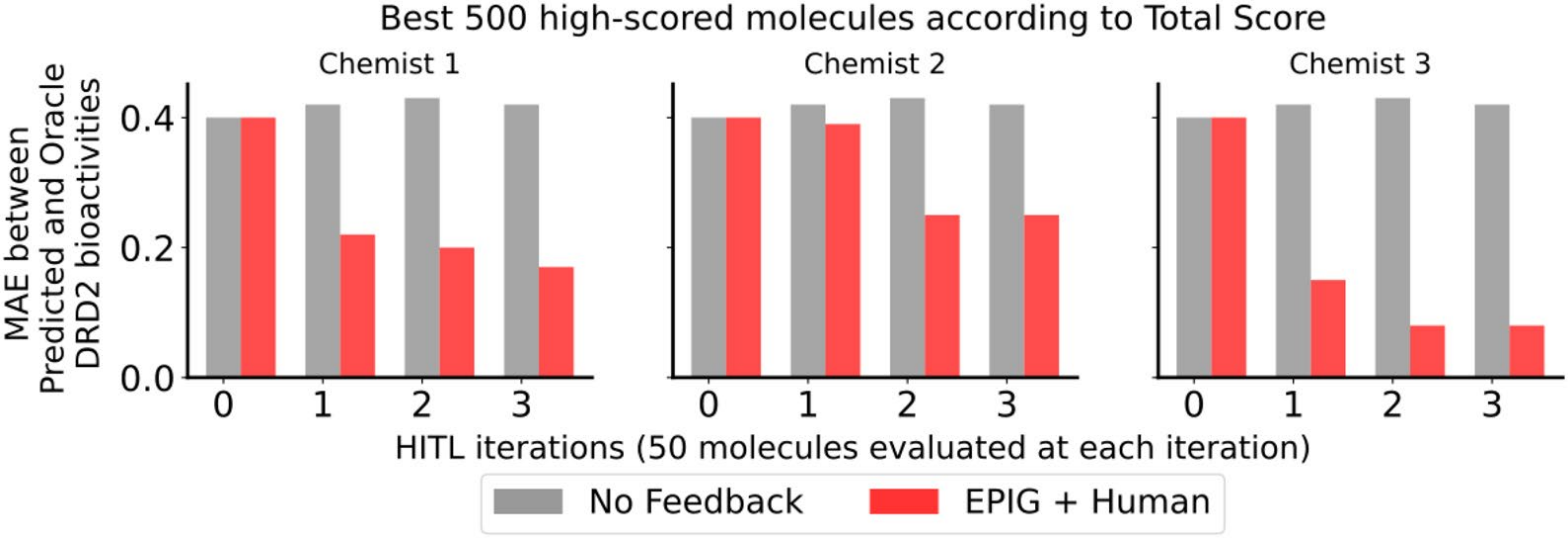
Evolution of the MAE between predicted and oracle DRD2 bioactivity scores during a multi-objective molecule generation run with (in red) and without (in gray) chemist intervention. EPIG was used for query selection. MAEs were computed on the top 500 high-scoring molecules across the three generation objectives

Across all three chemists, we observe an enhanced alignment between the mean predicted DRD2 bioactivity score and mean oracle score for the generated molecules throughout the process (Fig. 8). This indicates that incorporating chemist feedback on DRD2 predictions and updating the generative agent leads to exploring different regions of the chemical space where molecules are more likely to be bioactive according to the oracle (Additional file 1: Figure S10). This improved alignment is noted in comparison to scenarios where no feedback is solicited to update the DRD2 predictor, revealing a more pronounced gap between the predicted and oracle DRD2 bioactivities, and therefore the production of less optimal molecules.

**Fig. 9:**
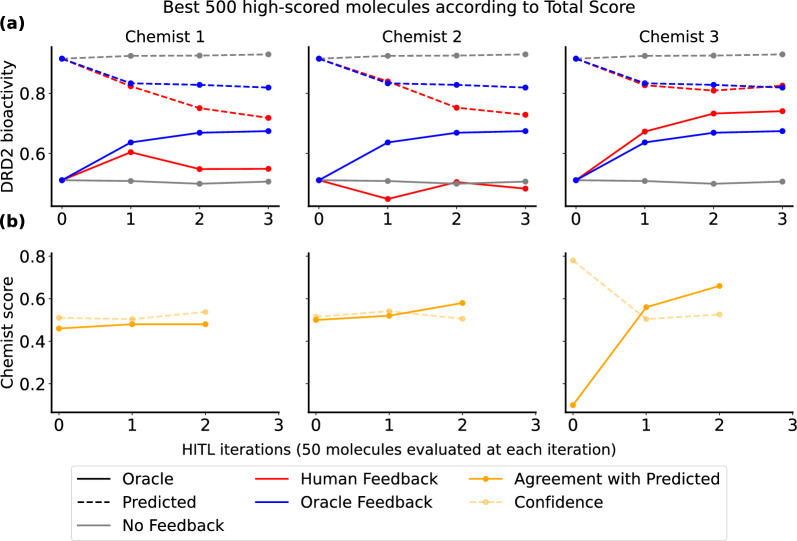
**a** Chemist intervention using EPIG significantly improves the alignment between predicted and oracle DRD2 bioactivity scores (in red) compared to no intervention (in gray). To compare the impact of real chemist intervention versus the oracle (as described in Eq. 11), we also report the DRD2 bioactivity scores obtained through optimizing the fine-tuned predictor (in blue). EPIG was used for selecting the queries for both chemists and the oracle. **b** Percentage agreement of chemist assessments with DRD2 bioactivity predictions (solid orange line) and their confidence scores (dashed orange line) across the top 500 high-scoring molecules from the multi-objective generation run

Regarding the enrichment of the top-scoring molecules in true positives, two out of the three chemist experiments resulted in improved enrichment compared to the “No Feedback” baseline (Fig. 9**a**). The most notable improvement was observed when interacting with chemist 3. Both the mean oracle score of the generated molecules and the percentage of agreement with the predicted DRD2 bioactivities have increased as a result of updating the predictor based on chemist 3 feedback, suggesting that the general knowledge of this chemist about DRD2 bioactivity better aligns with the true likelihood of DRD2 bioactivity (Fig. 9**b**). Consequently, inferring well-aligned feedback from chemist 3 leads to an improved understanding of the structure-activity relationship by the updated DRD2 predictor which in turn improves the task of designing new active candidates for DRD2.

We also investigated how each chemist experienced this exercise after its completion. While the goal of acquiring feedback was to calibrate the DRD2 bioactivity predictor and obtain molecules that are more likely to be active, chemists 1 and 2 had a different understanding. Chemist 1, who had never worked with the DRD2 target before and lacked specific knowledge about the structure-activity relationship, understood the instructions to assess how much they liked the molecule as a lead. Consequently, they aimed to select molecules that were synthesisable, stable, and with reasonable lipophilicity to maximize the chances of them being made and tested in a project. Similarly, chemist 2, who was also unfamiliar with the DRD2 dataset and binding site, did not fully grasp the intended focus on assessing the validity of the DRD2 bioactivity predictions. We noticed that this misunderstanding stemmed from the question displayed through the GUI during these HITL experiments, which was “How much would you prioritize this molecule as a DRD2 binder?”. This question did not clearly convey the emphasis on validating the DRD2 bioactivity predictions. For the third HITL experiment, we reformulated the question to “How strongly do you agree with this molecule being predicted as a DRD2 binder?” (Fig. 2) to better align with the goal of obtaining feedback on the predictor’s accuracy. Chemist 3 focused on known DRD2 actives to judge the plausibility of new proposed actives and also considered drug-likeness criteria. Unlike chemists 1 and 2, chemist 3 acknowledged a clear understanding of the goal, which explains why the third human experiment successfully led to the desired outcome. This highlights the importance of carefully designing user studies to enhance the efficiency of HITL approaches. Overall, all chemists found the interface easy to use and had a positive experience.

Furthermore, we investigated the enhancement of other metrics before and after querying the chemists. Table 1 displays the results for no feedback, as well as feedback from chemists 1 to 3 across different metrics from MOSES.

**Table 1:** Metrics calculated on the top 500 high-scoring molecules obtained from a multi-objective molecule generation where DRD2 bioactivity, hERG inactivity, and QED were optimized simultaneously (^†^Total Oracle score combines both DRD2 bioactivity and hERG inactivity oracles, as well as the QED score via Eq. 1. RO3 MolLogP is a lead-like filter which corresponds to the percentage of molecules that satisfy the condition MolLogP $< 3$)

Metric (mean)	No feedback	With feedback on DRD2 bioactivity		
		Chemist 1	Chemist 2	Chemist 3
DRD2 Predicted score	0.93	0.80 **	0.81 **	0.84 **
DRD2 Oracle score $\uparrow$	0.50	0.55	0.49	**0.74** **
Mean Absolute Error $\downarrow$	0.42	0.25 **	0.32 **	**0.10** **
QED score $\uparrow$	0.57	0.61 **	0.58	**0.71** **
hERG inactivity score $\uparrow$	0.91	0.88	0.90	0.82
Total Oracle score^†^ $\uparrow$	0.68	0.69	0.67	**0.77** **
Internal Diversity $\uparrow$	0.47	0.41	0.45	0.44
RO3 MolLogP $\uparrow$	0.70	0.54 **	**0.79** **	0.66
SA score $\downarrow$	3.04	**2.75** **	**2.82** **	3.08
Novelty $\uparrow$	1.0	1.0	1.0	1.0
Uniqueness $\uparrow$	1.0	1.0	1.0	1 .0

Values that significantly differ from the "No Feedback" baseline (ANOVA *p*-value < 0.01) are marked with the superscript **. Values in bold correspond to the most performant methods in comparison with the "No Feedback" baseline

Overall, the inclusion of HITL-AL leads to a notable improvement in the outcomes of molecule generation across a various range of metrics. In comparison to when no chemist feedback is queried, the generated high-scoring molecules exhibit a significantly higher likelihood of being active against the target protein, as observed in interactions with chemist 3 (ANOVA *p*-value: $2.10^{-146} < 0.05$). Additionally, molecules show improved properties, including better SA (the lower the SA score the easier to synthesize a molecule), as evidenced in interactions with chemists 1 (ANOVA *p*-value: $9.10^{-91} < 0.05$) and 2 (ANOVA *p*-value: $5.10^{-56} < 0.05$), who focused their feedback on prioritizing molecules based on their SA, as well as higher QED, as a result of interacting with chemist 3 (ANOVA *p*-value: $4.10^{-94} < 0.05$). Notably, enabling HITL-AL with different chemists during a goal-oriented molecule generation process leads to a better alignment between predicted and oracle scores for the top generated molecules (represented by lower mean absolute errors, ANOVA *p*-values of $5.10^{-156}$, $7.10^{-70}$ and $1.10^{-250}$ from interacting with each chemist respectively). An interesting future direction would be to use an ensemble of predictive models updated from different chemists to score the generated molecules. Additionally, it does not compromise the final novelty and uniqueness of the generated molecules. The observed decrease in internal diversity compared to no feedback is not deemed significant (ANOVA *p*-values of 0.22, 0.98 and 0.81 from interacting with each chemist respectively). These results underscore the importance of incorporating informed chemist feedback to augment ML predictions when used to guide molecule generation processes, ensuring more grounded predictions and leading to more meaningful and practical generation results.

## Conclusion

In this study, we have introduced an innovative approach that leverages human–machine collaboration in the form of HITL-AL to fine-tune property predictors for molecule generation, thereby enhancing their generalizability and alignment with real-world applications. This methodology proves especially valuable in scenarios where limited labeled data is available for building generalizable proxies for molecule generation, and where pre-training the molecule generator or optimizing training data similarity might unduly constrain the diversity and creativity of the generated molecules.

Our proposed approach is particularly relevant in contexts such as exploring novel protein receptors, where existing experimental data may be scarce, yet the need for finding new and potentially impactful ideas is pressing. By integrating expert evaluations into the molecule generation process, we not only enhance the predictive capabilities of our proxy models but also ensure that the generated molecules are more meaningful and better aligned with the complexities of real-world scenarios.

A critical component of our approach is the use of an oracle evaluator, and in real-world applications, human experts may need to act as this oracle. The number of cycles of HITL-AL required for effective model refinement depends on several factors, including the quality of the initial dataset and the expertise of the evaluators. High-quality ground-truth data and feedback can significantly reduce the number of cycles needed. Additionally, setting clear convergence criteria, such as monitoring the stabilization of expert feedback patterns, or the reduction of prediction errors on an external validation set, can also help determine when to stop the AL process. Based on our findings, a range of 3 to 5 AL cycles often serves as a reasonable starting point, but the exact number should be tailored to the specific project requirements.

In summary, our methodology bridges the gap between generative AI for chemistry and human intuition. By incorporating expert evaluation feedback alongside existing experimental data, we create a closed-loop system wherein generative models continuously refine their predictions in response to real-world feedback, fostering greater trust in the final outcomes. As the field advances towards closed-loop discovery platforms, there is a growing trend of integrating automated iterative systems that cycle through hypothesis generation, experimentation and analysis. These platforms leverage AI to continuously generate and test new ideas, then refine models based on insights gained from the analysis. Our approach represents a significant step forward in combining the strengths of generative AI and human expertise, paving the way for more robust processes that better serve real-world applications.

**Table 2:** Metrics calculated for the final selections of high-scoring molecules according to the LogP predictor (Score > 0.5)

Metric	No Feedback	Feedback ($T = 10$)			
		EPIG ($\sigma _{\epsilon } = 5.0$)	Uncertainty ($\sigma _{\epsilon } = 5.0$)	Greedy ($\sigma _{\epsilon } = 5.0$)	Random ($\sigma _{\epsilon } = 5.0$)
Number of molecules	124.44 ± 1.34	125.38 ± 1.58	126.22 ± 1.40	125.89 ± 1.52	124.78 ± 1.62
MAE Oracle-Pred. $\downarrow$	2.15 ± 0.24	1.35 ± 0.16 **	**1.29** ± **0.21** **	1.42 ± 0.19 **	1.91 ± 0.50
Internal Diversity $\uparrow$	0.85 ± 0.01	0.84 ± 0.01	0.84 ± 0.01 *	0.84 ± 0.01	**0.85 ± 0.01**
SA $\downarrow$	3.12 ± 0.15	2.80 ± 0.12 **	**2.78** ± **0.15** **	2.83 ± 0.06 **	3.04 ± 0.16
QED $\uparrow$	0.52 ± 0.03	0.45 ± 0.03 **	0.44 ± 0.03 **	0.46 ± 0.04 **	0.51 ± 0.05
Novelty $\uparrow$	1.0 ± 0.0	1.0 ± 0.0	1.0 ± 0.0	1.0 ± 0.0	1.0 ± 0.0
Uniqueness $\uparrow$	1.0 ± 0.0	1.0 ± 0.0	1.0 ± 0.0	1.0 ± 0.0	1.0 ± 0.0
Frag Gen-Train $\uparrow$	0.70 ± 0.05	0.85 ± 0.05 **	0.88 ± 0.03 **	**0.88** ± **0.04** **	0.86 ± 0.04 **
SNN Gen-Train $\uparrow$	0.23 ± 0.01	0.26 ± 0.01 **	**0.27** ± **0.01** **	0.26 ± 0.01 **	0.25 ± 0.01 **
FCD Gen-Train $\downarrow$	35.64 ± 1.31	**30.07** ± **1.07** **	30.88 ± 1.69 **	31.81 ± 1.07 **	32.30 ± 1.62 **
Frag Gen-Queries $\uparrow$	-	**0.96** ± **0.02**	0.95 ± 0.02	0.94 ± 0.02	0.92 ± 0.03
SNN Gen-Queries $\uparrow$	-	**0.27** ± **0.01**	**0.27** ± **0.02**	0.26 ± 0.00	0.25 ± 0.01
FCD Gen-Queries $\downarrow$	-	27.12 ± 1.03	27.45 ± 0.87	27.23 ± 1.07	**26.47 ± 1.37**

For all metrics, we report the mean and standard deviation across 10 different replicates of each experimental run. Up and down arrows indicate the expected direction of improvement for each metric. One-sided ANOVA tests were applied for statistical significance assessments, and performance significance with respect to the “No Feedback” baseline is marked with * (if *p*-value $< 0.05$) or ** (if *p*-value $< 0.01$)

Metric values in bold correspond to the most performant methods

**Table 3:** Metrics calculated for the final selections of high-scoring molecules according to the DRD2 bioactivity predictor ($> 0.5$), mono-objective optimization

Metric	No Feedback	Feedback ($T = 10$)			
		EPIG ($\sigma _{\epsilon } = 0.3$)	Uncertainty ($\sigma _{\epsilon } = 0.3$)	Greedy ($\sigma _{\epsilon } = 0.3$)	Random ($\sigma _{\epsilon } = 0.3$)
Number of molecules	121.00 ± 1.41	97.63 ± 6.04	88.00 ± 12.39	85.11 ± 22.53	98.43 ± 11.13
MAE Oracle-Pred. $\downarrow$	$0.61 \pm 0.02$	$0.23 \pm 0.05$ **	**0.14** ± **0.05** **	$0.31 \pm 0.04$ **	$0.15 \pm 0.04$ **
Internal Diversity $\uparrow$	$0.70 \pm 0.01$	$0.60 \pm 0.03$ **	$0.60 \pm 0.03$ **	**0.65** ± **0.06** *	$0.57 \pm 0.02$ **
SA $\downarrow$	**3.36** ± **0.09**	$3.58 \pm 0.44$	$3.63 \pm 0.31$ *	$3.63 \pm 0.34$ *	$3.91 \pm 0.57$ *
QED $\uparrow$	$0.41 \pm 0.03$	**0.60** ± **0.08** **	$0.54 \pm 0.10$ **	$0.51 \pm 0.08$ **	$0.50 \pm 0.06$ **
Novelty $\uparrow$	1.0 $\pm 0.0$	1.0 $\pm 0.0$	1.0 $\pm 0.0$	1.0 $\pm 0.0$	1.0 $\pm 0.0$
Uniqueness $\uparrow$	1.0 ± 0.0	1.0 ± 0.0	1.0 ± 0.0	1.0 ± 0.0	1.0 ± 0.0
Frag Gen-Train $\uparrow$	0.95 $\pm 0.01$	$0.90 \pm 0.10$	$0.85 \pm 0.20$	$0.64 \pm 0.21$ **	$0.90 \pm 0.18$
SNN Gen-Train $\uparrow$	$0.41 \pm 0.01$	$0.49 \pm 0.02$ **	$0.52 \pm 0.02$ **	$0.46 \pm 0.05$ *	**0.52** ± **0.03** **
FCD Gen-Train $\downarrow$	39.23 ± 2.03	$37.17 \pm 3.34$	$\textbf{36.19} \pm \textbf{3.85}$	$40.96 \pm 5.46$	$38.46 \pm 3.97$
Frag Gen-Queries $\uparrow$	-	**0.97** ± **0.10**	$0.91 \pm 0.12$	$0.67 \pm 0.17$	**0.97** ± **0.05**
SNN Gen-Queries $\uparrow$	-	**0.54** ± **0.03**	$0.50 \pm 0.01$	$0.49 \pm 0.07$	**0.54** ± **0.02**
FCD Gen-Queries $\downarrow$	-	$11.99 \pm 2.61$	$14.80 \pm 4.90$	$23.77 \pm 12.06$	**10.25** ± **1.59**

For all metrics, we report the mean and standard deviation across 10 different replicates of each experimental run. Up and down arrows indicate respectively whether each performance metric is expected to increase or decrease. One-sided ANOVA tests were applied for statistical significance assessments, and performance significance with respect to the “No Feedback” baseline is marked with * (if *p*-value $< 0.05$) or ** (if *p*-value $< 0.01$)

Metric values in bold correspond to the most performant methods

**Table 4:** Metrics calculated for the final selections of high-scoring molecules according to the DRD2 bioactivity predictor ($> 0.5$), multi-objective optimization

Metric	No Feedback	Feedback ($T = 10$)			
		EPIG ($\sigma _{\epsilon } = 0.3$)	Uncertainty ($\sigma _{\epsilon } = 0.3$)	Greedy ($\sigma _{\epsilon } = 0.3$)	Random ($\sigma _{\epsilon } = 0.3$)
Number of molecules	104.11 ± 7.72	65.75 ± 17.23	63.14 ± 20.52	77.56 ± 15.37	88.11 ± 22.54
MAE Oracle-Pred. $\downarrow$	$0.42 \pm 0.01$	**0.19** ± **0.03** **	0.21 ± 0.05 **	$0.25 \pm 0.02$ **	**0.19** ± **0.01** **
Internal Diversity $\uparrow$	$0.62 \pm 0.01$	$0.60 \pm 0.01$ **	$0.59 \pm 0.02$ **	**0.62** ± **0.01**	$0.58 \pm 0.02$ **
SA $\downarrow$	$3.26 \pm 0.07$	$3.28 \pm 0.08$	$3.30 \pm 0.12$	**3.23** ± **0.08**	$3.26 \pm 0.11$
QED $\uparrow$	$0.61 \pm 0.02$	0.64 ± 0.05	$0.63 \pm 0.05$	**0.67** ± **0.03** **	$0.61 \pm 0.03$
Novelty $\uparrow$	$1.0 \pm 0.0$	$1.0 \pm 0.0$	$1.0 \pm 0.0$	$1.0 \pm 0.0$	$1.0 \pm 0.0$
Uniqueness $\uparrow$	1.0 ± 0.0	1.0 ± 0.0	1.0 ± 0.0	1.0 ± 0.0	1.0 ± 0.0
Frag Gen-Train $\uparrow$	$0.92 \pm 0.02$	**0.96** ± **0.02** **	**0.96** ± **0.01** **	$0.92 \pm 0.02$	$0.90 \pm 0.18$
SNN Gen-Train $\uparrow$	$0.47 \pm 0.01$	$0.50 \pm 0.01$ **	$0.51 \pm 0.02$ **	$0.48 \pm 0.02$	**0.54** ± **0.02** **
FCD Gen-Train $\downarrow$	40.53 ± 1.39	$39.17 \pm 1.85$	$38.86 \pm 2.26$	$40.33 \pm 1.47$	**38.46** ± **3.97**
Frag Gen-Queries $\uparrow$	-	**0.97** ± **0.01**	$0.96 \pm 0.02$	$0.91 \pm 0.02$	**0.97** ± **0.05**
SNN Gen-Queries $\uparrow$	-	**0.52** ± **0.02**	$0.45 \pm 0.01$	$0.48 \pm 0.01$	$0.47 \pm 0.01$
FCD Gen-Queries $\downarrow$	-	**15.86** ± **2.00**	$17.42 \pm 2.27$	$29.46 \pm 3.77$	$32.33 \pm 3.91$

For all metrics, we report the mean and standard deviation across 10 different replicates of each experimental run. One-sided ANOVA tests were applied for statistical significance assessments, and performance significance with respect to the “No Feedback” baseline is marked with * (if *p*-value $< 0.05$) or ** (if *p*-value $< 0.01$)

Metric values in bold correspond to the most performant methods

However, it is crucial to acknowledge the inherent limitations of our method to provide a more comprehensive understanding of the scope and applicability of our approach in real-world settings. The effectiveness of our approach relies heavily on the availability and expertise of human collaborators, introducing the potential for subjectivity and variability in feedback interpretation. Balancing these diverse perspectives while ensuring consistency in feedback poses a significant challenge that warrants careful consideration. In this work, we addressed this by downweighting uncertain human inputs during the fine-tuning of property predictors. Furthermore, the integration of human feedback into the generation process introduces computational complexities and overhead, potentially impacting scalability and efficiency, particularly in large-scale drug discovery projects. Ethical considerations surrounding data privacy, transparency, and bias mitigation are also paramount to ensure the responsible and equitable utilization of technology. Future work could focus on scaling up the approach to include multiple experts or automated agents in the loop, allowing for parallelized feedback cycles and improved scalability in high-throughput settings.

## Supplementary Information

Additional file 1.

## Figures and Tables

**Fig. 10:**
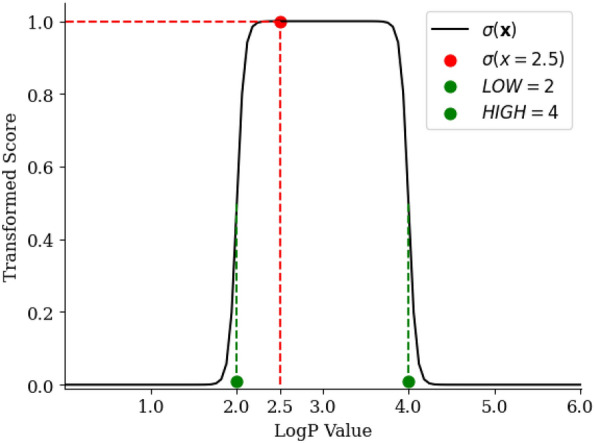
Double sigmoid transformation function used in use-case 1 experiment to map predicted LogP values to [0, 1] scores

## Data Availability

Code and data for reproducing the simulations are available at https://github.com/yasminenahal/hitl-al-gomg. DRD2 and hERG bioactivity oracles can be downloaded from https://huggingface.co/yasminenahal/hitl-al-gomg-simulators/tree/main.

## Appendix A Training the random forest models

In Eq. 2, the set of parameters $\varvec{\theta }$ represent the number of nodes, the decision features and their corresponding threshold values used by each decision tree when making a decision about a new input. These parameters are determined during the RF training process, and depend on the training data ${\mathcal {D}}_{\text {round}}$ and the loss criterion being optimized.

During training, bootstrap sampling is used which involves creating multiple subsets of training data by randomly drawing data points with replacement from the original dataset ${\mathcal {D}}_{\text {round}}$. Each tree in the RF is then trained on one of these bootstrap samples. The use of sample weights $w_i$ and $u_t$ during bootstrapping allows certain data points to be sampled with a higher probability than others. During the tree (re-)building process, when selecting new data points for each node split, the algorithm can consider the sample weights assigned during bootstrapping to ensure that the decision trees are constructed with attention to the importance of each data point based on its weight. By assigning lower weights to uncertain expert labels, the RF can give less emphasis to those newly introduced data points during model updates.

The loss criterion being optimized also plays a crucial role during model updates. In the binary classification case, the Gini impurity of tree nodes is used to guide the tree (re-)building process. It is defined as:

A1 $$\begin{aligned} \operatorname {Gini}(\operatorname {node}) = 1 - \sum _{y \in \{0,1\}} p(y \mid \operatorname {node})^2 \end{aligned}$$

where $y \in \{0,1\}$ is the class label and $p(y \mid \operatorname {node})$ is the proportion of samples associated with class label *y* among all samples in the node.

This criterion is used to guide the construction of decision trees during the training process of a RF. When splitting a node into two child nodes, the Gini impurity is used to evaluate the quality of the split. The Gini impurity for a split is a weighted average of the Gini impurity of the child nodes, where the weights are proportional to the number of samples in each child node. For a binary split on feature *d* at threshold *t*, the Gini impurity is calculated as:

A2 $$\begin{aligned} \operatorname {Gini}_{\text {split}}(d,t) = \frac{N_{\text {left}}}{N} \operatorname {Gini}(\text {left}) + \frac{N_{\text {right}}}{N} \operatorname {Gini}(\text {right}) \end{aligned}$$

Here, $N_{\text {left}}$ and $N_{\text {right}}$ are the number of samples in the left and right child nodes after the split, respectively. $N_{\text {total}}$ is the total number of samples in the current node. $\operatorname {Gini}(\text {left})$ and $\operatorname {Gini}(\text {right})$ are the Gini impurities of the left and right child nodes, respectively.

In the regression case, the mean squared error (MSE) is commonly used as the criterion to measure the quality of a split in decision trees. The MSE for a given node is defined as:

A3 $$\begin{aligned} \operatorname {MSE}(\operatorname {node}) = \frac{1}{N} \sum _{i=1}^N \left( y_i - \bar{y}_{\operatorname {node}}\right) ^2 \end{aligned}$$

where *N* is the total number of samples in the current node, $y_i$ represents the target value for the *i*th data point in the node, and $\bar{y}_{\operatorname {node}} = \frac{1}{N} \sum _{i=1}^N y_i$ represents the mean of all target values in the node.

For a given split that divides the current node into two child nodes, the total MSE reduction $\operatorname {MSE}_{\operatorname {reduction}}$ is calculated as the difference between the MSE of the parent node and the weighted sum of the MSEs of the child nodes:

A4 $$\begin{aligned} \operatorname {MSE}_{\operatorname {reduction}} = \operatorname {MSE}(\operatorname {node}) - \left( \frac{N_{\text {left}}}{N} \operatorname {MSE}(\text {left}) + \frac{N_{\text {right}}}{N} \operatorname {MSE}(\text {right})\right) \end{aligned}$$

The algorithm selects the split that maximizes the MSE reduction at each node. This way, it aims to find splits that minimize the variance of target values within each node, leading to a tree that provides an overall good fit to the target values in the dataset.

## Appendix B Predictive performance metrics

Pearson correlation coefficient: measures the proportion of variance in the dependent variable that is predictable from the independent variables used in a regression model.

B5 $$\begin{aligned} \rho = \frac{\sum _{i=1}^{N} (y_i - {\bar{y}})({\hat{y}}_i - \bar{{\hat{y}}})}{\sqrt{\sum _{i=1}^{N} (y_i - {\bar{y}})^2 \sum _{i=1}^{N} ({\hat{y}}_i - \bar{{\hat{y}}})^2}} \end{aligned}$$

where $y_i$ and ${\hat{y}}_i$ are the true and predicted values for the *i*th observation, and $\bar{y}$ and $\bar{{\hat{y}}}$ their respective means.

Mean Absolute Error (MAE): average absolute differences between predicted and true values, providing a measure of prediction accuracy.

B6 $$\begin{aligned} \text {MAE} = \frac{1}{N} \sum _{i=1}^{N} \left| y_i - {\hat{y}}_i \right| \end{aligned}$$

Receiver Operating Characteristic - Area Under the Curve (ROC AUC): measures a classifier’s ability to distinguish between positive and negative classes across different probability thresholds.

B7 $$\begin{aligned} \text {ROC AUC} = \int _{0}^{1} \text {TPR}(\text {FPR}^{-1}(t)) \, dt \end{aligned}$$

Precision-Recall Area Under the Curve (PR AUC): measures a classifier’s ability to balance precision and recall across different probability thresholds.

B8 $$\begin{aligned} \text {PR AUC} = \int _{0}^{1} \text {Precision}(\text {Recall}^{-1}(t)) \, dt \end{aligned}$$

Matthews Correlation Coefficient (MCC): measures the quality of binary classifications, considering true positives (TP), true negatives (TN), false positives (FP) and false negatives (FN).

B9 $$\begin{aligned} \text {MCC} = \frac{\text {TP} \times \text {TN} - \text {FP} \times \text {FN}}{\sqrt{(\text {TP} + \text {FP})(\text {TP} + \text {FN})(\text {TN} + \text {FP})(\text {TN} + \text {FN})}} \end{aligned}$$

## Appendix C The double sigmoid function

The double sigmoid function is defined as

C10 $$\begin{aligned} \sigma (x, \varvec{\theta }_{\sigma }) = \frac{10^{\alpha _1 x}}{10^{\alpha _1 x}+10^{\alpha _1 LOW}} - \frac{10^{\alpha _2 x}}{10^{\alpha _2 x}+10^{\alpha _2 HIGH}}, \end{aligned}$$

where $\varvec{\theta }_{\sigma } = \{\alpha _1, \alpha _2, LOW, HIGH\}$. Parameters *LOW* and *HIGH* define the target interval for $x \in {\mathbb {R}}$ and which are set to 2 and 4 respectively for the purpose of generating molecules with LogP in [2, 4]. Parameters $\alpha _1$ and $\alpha _2$ control the steepness of the rising and descending edge of the double sigmoid, respectively. Both are set to 10. Figure 10 illustrates this function and one realization at $x = 2.5$.

## Appendix D oracles

Penalized LogP oracle: Following Gómez-Bombarelli et al. [26], we use the octanol-water partition coefficient (LogP), as implemented in RDKit, minus the synthetic accessibility score [37] and the number of long cycles. This results in a penalized LogP that we use as an oracle which calculation is provided by the TDC benchmarking python package [38].

DRD2 bioactivity oracle: We use a RFC that consists of 300 estimators and a maximum depth of 20, trained using Scikit-learn on DRD2 bioactivity data retrieved from the ExCAPE database [31]. This dataset holds DRD2 bioactivity measures (pIC50) from PubChem [39] and ChEMBL [24]. Binary bioactivity labels were generated by setting a cutoff value of pIC50 at 7.3, such as molecules with pIC50 above the cutoff are labelled as active. As the DRD2 dataset initially contained too many active compounds, we sampled additional random molecules from ChEMBL, which we considered as additional inactive compounds. Only molecules from ChEMBL that had a molecular weight below 800 and a similarity between 0.1 and 0.5 to any molecules in the original DRD2 dataset set were considered. Molecules were sampled until we had reached a percentage of actives below 1%. The dataset was split into training and test sets using a scaffold split. Training samples were weighted to create a class balance (class_weight=”balanced”). Model performance on the test set is reported in Additional file 1: Table S1. The predicted positive class probabilities by this model are used as ground truth values.

hERG bioactivity oracle: We use a RFC consisting of 300 estimators and a maximum depth of 20, trained using Scikit-learn on hERG bioactivity data retrieved from ExCAPE where bioactivities are measured in terms of pIC50. Molecules with a pIC50 $> 5.0$ were labelled as active, and the remaining were labelled as inactive. Randomly sampled inactive molecules were removed from the dataset to ensure that 10% of the training set are active molecules. The dataset was split into training and test sets using a scaffold splitting strategy. To ensure a better balance between sampling from both active and inactive classes during the training process, all training samples were weighted based on their labels. This strategy is akin to the class_weight=”balanced” parameter implementated in Scikit-learn, where it automatically adjusts the weights inversely proportional to class frequencies in the input data. The predicted positive class probabilities by this model are used as ground truth values.

## Appendix E Molecule generation metrics

The following metrics are provided by the benchmarking platform MOSES [36] and were used to assess the quality, diversity and novelty of generated molecules.

Quantitative Estimation of Drug-likeness (QED): a score within [0, 1] estimating how likely a molecule is a viable candidate for a drug. QED is meant to capture the abstract notion of esthetics in medicinal chemistry [33].

Synthetic Accessibility score (SA): based on a combination of molecular fragments contributions [37], it estimates how hard (10) or easy (1) it is to synthesize a given molecule, so the lower the SA score the better.

Fragment similarity (Frag): compares distributions of BRICS fragments [40], denoted as *F*, between a generated molecular set *G* and a reference molecular set *R*. The metric is defined as a cosine similarity and its limits are [0, 1]. If molecules in both sets have similar fragments, the metric is large. If some fragments are over- or underrepresented in the generated set, the metric will be lower.

E11 $$\begin{aligned} \operatorname {Frag}(G,R) = \frac{\sum _{f \in F} [c_f(G) c_f(R)]}{\sqrt{\sum _{f \in F} c_f^2(G)} \sqrt{\sum _{f \in F} c_f^2(R)}} \end{aligned}$$

Similarity to a nearest neighbor (SNN): computes average Tanimoto similarity between fingerprints of a molecule from the generated set *G* and its nearest neighbor molecule in the reference set *R*. The metric is limited by [0, 1]. If generated molecules are far from the manifold of the reference set, the similarity to the nearest neighbor will be low. In MOSES, Tanimoto similarity is based on standard Morgan ECFP with radius 2 and 1024 bits computed using RDKit.

E12 $$\begin{aligned} \operatorname {SNN}(G,R) = \frac{1}{|G|} \sum _{m_G \in G} \operatorname {max}_{m_R \in R} \operatorname {Tanimoto}(m_G, m_R) \end{aligned}$$

Fréchet ChemNet Distance (FCD) [41] **:** calculated using activations of the penultimate layer of a deep neural network, ChemNet, trained to predict biological activities of drugs. These activations are calculated for canonical SMILES representations and capture both chemical and biological properties of the molecules. The lower FCD the better, and it cannot be negative.

E13 $$\begin{aligned} \operatorname {FCD}(G,R) = ||\mu _G - \mu _R||^2 + \operatorname {tr} \left[ \Sigma _G + \Sigma _R - 2 \sqrt{\Sigma _G \Sigma _R} \right] \end{aligned}$$

where $\mu _G$ and $\mu _R$ are mean vectors and $\Sigma _G$ and $\Sigma _R$ are covariance matrices of activations for molecules from sets *G* and *R* respectively.

Internal Diversity (IntDiv_*p*_) [42] **:** assesses the chemical diversity within a generated molecular set *G*. A higher value of this metric corresponds to higher diversity in the generated set. The metric is limited by [0, 1]. We mainly consider $\operatorname {IntDiv}_1(G)$ in this work.

E14 $$\begin{aligned} \operatorname {IntDiv}_p(G) = 1 - \sqrt{\frac{1}{|G|} \sum _{m_1,m_2 \in G} \operatorname {Tanimoto}(m_1,m_2)^{p}} \end{aligned}$$

Novelty: fraction of the generated molecules that are not present in the training set.

Uniqueness: fraction of the generated molecules that are unique. This metric checks that the model does not collapse to producing only a few typical molecules.

## Abbreviations

AI: Artificial intelligence
AL: Active learning
DRD2: Dopamine receptor D2
EPIG: Expected predictive information gain
hERG: Human ether-à-go-go-related gene
HITL: Human-in-the-loop
MAE: Mean absolute error
ML: Machine learning
LogP: Partition coefficient
RF: Random forest
RFC: Random forest classifier
RFR: Random forest regressor
RL: Reinforcement learning
RNN: Recurrent neural network
SMILES: Simplified molecular-input line-entry system
QED: Quantitative estimate of drug-likeness
QSAR: Quantitative structure-activity relationship
QSPR: Quantitative structure–property relationship
