# Supplementary material for: Human-in-the-loop active learning for goal-oriented molecule generation
Source: J Cheminform. 2024 Dec 9;16:138. doi: 10.1186/s13321-024-00924-y (PMC11629536; doi:10.1186/s13321-024-00924-y)
Supplement: Supplementary file 1 — Additional file 1. [file 13321_2024_924_MOESM1_ESM.pdf]

Additional file for Human-in-the-loop active learning for goal-oriented molecule generation

Yasmine Nahal[1,4] Janosch Menke[2] Julien Martinelli[3]
Markus Heinonen[1] Mikhail Kabeshov[4] Jon Paul Janet[4]
Eva Nittinger[5] Ola Engkvist[2,4*] Samuel Kaski[1,6,*]

[1]Department of Computer Science, Aalto University, Espoo, Finland
[2]Department of Computer Science and Engineering, Chalmers University of Technology, Gothenburg, Sweden
[3]Inserm Bordeaux Population Health, Vaccine Research Institute, Université de Bordeaux, Inria Bordeaux Sud-ouest, France
[4]Molecular AI, Discovery Sciences, BioPharmaceuticals R&D, AstraZeneca, Gothenburg, Sweden
[5]Medicinal Chemistry, Research and Early Development, Respiratory and Immunology (R&I), BioPharmaceuticals R&D, AstraZeneca, Gothenburg, Sweden
[6]Department of Computer Science, University of Manchester, Manchester, United Kingdom

Initial predictor training and performance assessment

Dataset	Actives	Inactives	Total	ROC AUC (Train/Test)	PR AUC (Train/Test)	MCC (Train/Test)
hERG	3800	34200	38000	0.99/0.96	0.99/0.838	0.96/0.761
DRD2	1039	100000	101039	1.00/0.99	0.97/0.63	0.83/0.59
Reduced DRD2	62	178	240	1.00/-	1.00/-	1.00/-

Table S1: Dataset specifications used for QSAR modelling and predictive performance on holdout test sets, prior to deployment.

*These authors contributed equally.

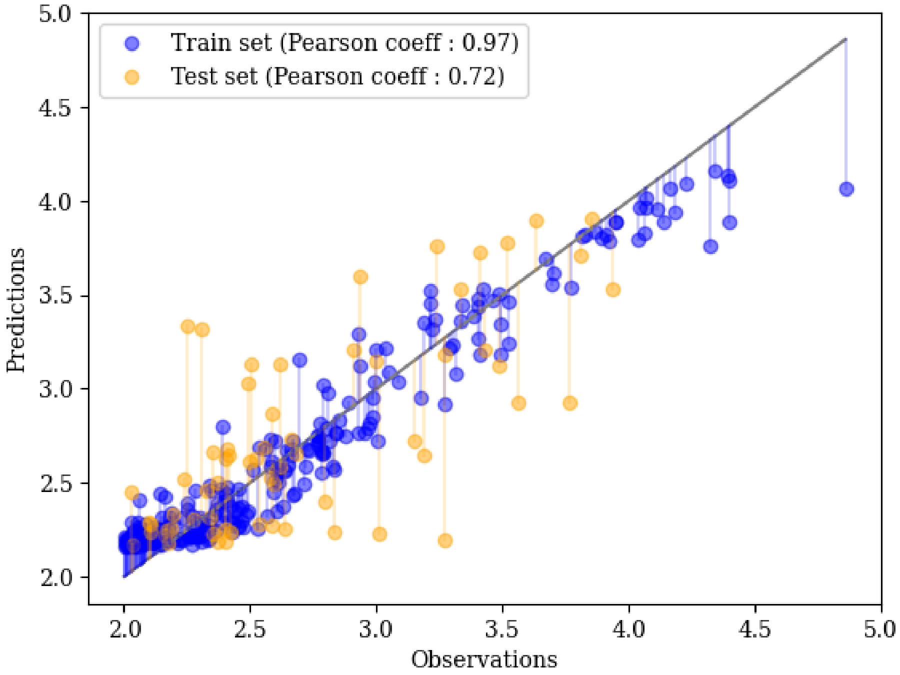

Figure S1: Linear correlation plot of predicted vs. actual LogP values for the initial dataset $\mathcal{D}_0$

Metis settings for the human experiments

```yaml
tutorial: false
debug: false
wandb: false
max_iterations: 3
innerloop_iterations: 5
activity_label: "DRD2"
introText: "We are interested in the design of an new binder 
    for the Dopamine receptor D2. We have identified two key 
    properties:"
propertyLabels:
  "DRD2 Activity": "raw_DRD2"
  "hERG Activity": "raw_herg"
data:
  initial_path: "../data/scaffold_memory_oracle_truth.csv"
  path: "../data/scaffold_memory.csv"
  selection_strategy: "epig"
  num_molecules: 10
  run_name: "chemist3_final"
ui:
  show_atom_contributions: false
  show_reference_molecules: true
  tab:
    render: false
    tab_names: ["General","DRD2"]
  navigationbar:
    sendButton:
      render: true
    editButton:
      render: true
    compareButton:
      render: false
  general:
```

```yaml
      render: true
      slider: true
    substructures:
      render: false
      liabilities:
        ugly:
          name: "Ugly"
          color: "#ff7f7f"
        tox:
          name: "Toxicity"
          color: "#51d67e"
        stability:
          name: "Stability"
          color: "#eed358"
        like:
          name: "Good"
          color: "#9542f5"
    global_properties:
      render: false
      liabilities: [
                "Solubility",
                "Lipophilicity",
                "Plasma␣Proteinbinding",
                "Synthetic␣Accessibility",
                "Permeability",
                "hERG",
                "Too␣Big",
                "Too␣Small",
            ]
interactive_model:
    oracle_score: false  # use oracle score or user model to
        update
    weight: "pseudo_confidence"
    use_human_component: false
    oracle_path: "reinvent_connect/input_files/drd2.pkl"
    model_path:
        "reinvent_connect/input_files/initial_qsar_model.pkl"
    training_data_path: "../data/qsar_data_scored_by_oracle.csv"
    ECFP:
      bitSize: 2048
      radius: 3
      useCounts: true
```

Listing S1: YAML file given as input to Metis for running the experiment with Chemist 3

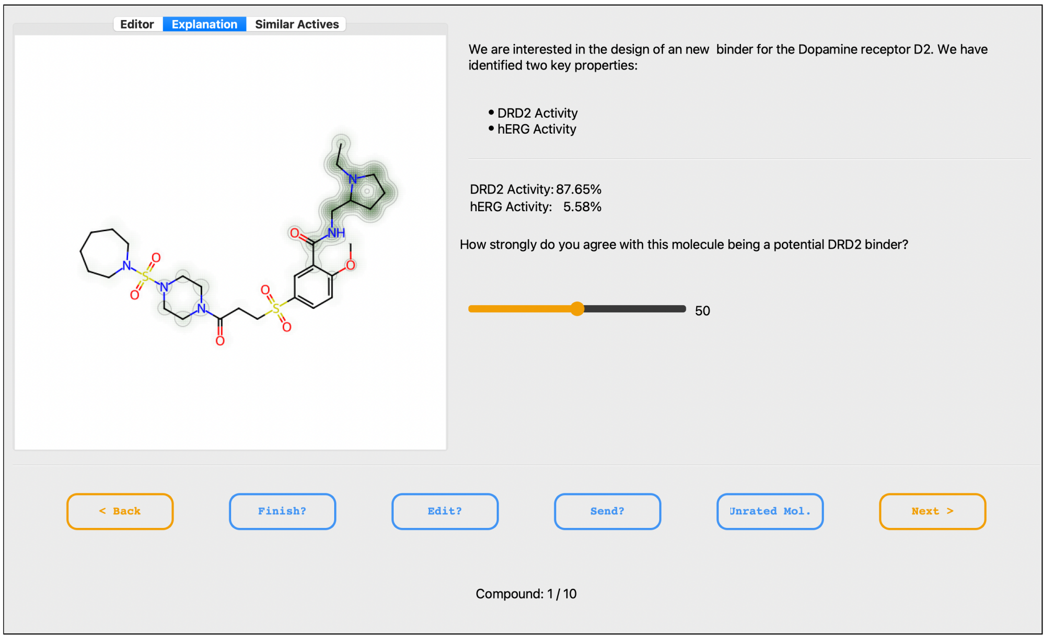

Figure S2: "Explanation" window on the GUI displaying visual explanations for individual DRD2 bioactivity predictions for the selected molecules via the EPIG acquisition strategy.

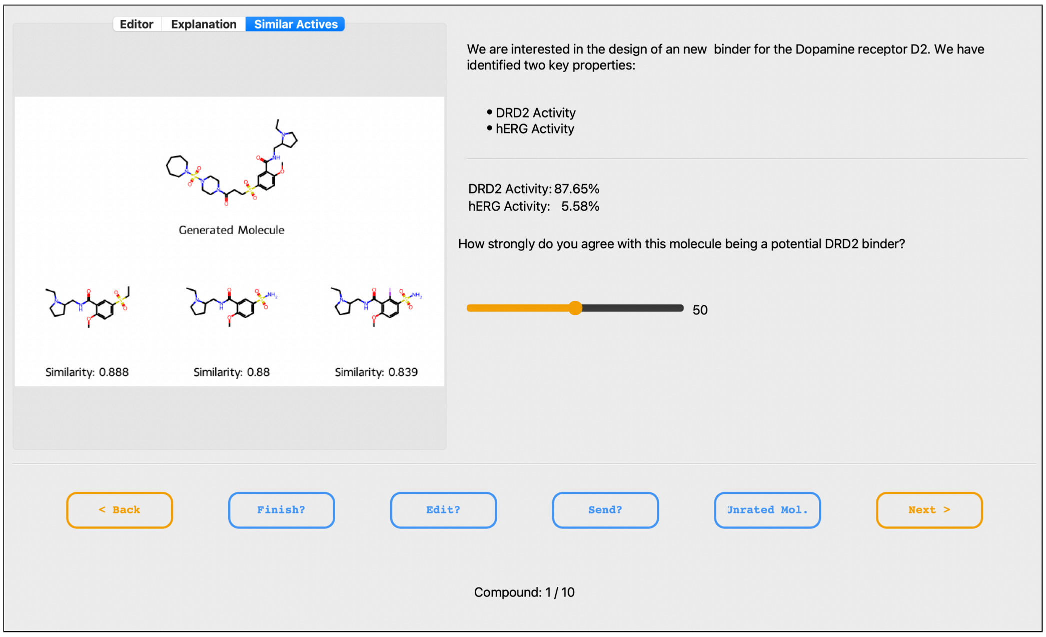

Figure S3: "Similar Actives" window on the GUI displaying the most similar active molecules already available in the initial training set of the DRD2 predictor for each of the selected molecules via the EPIG acquisition strategy. Molecular similarity is computed based on MACCS keys.

Additional results for the simulated experiments

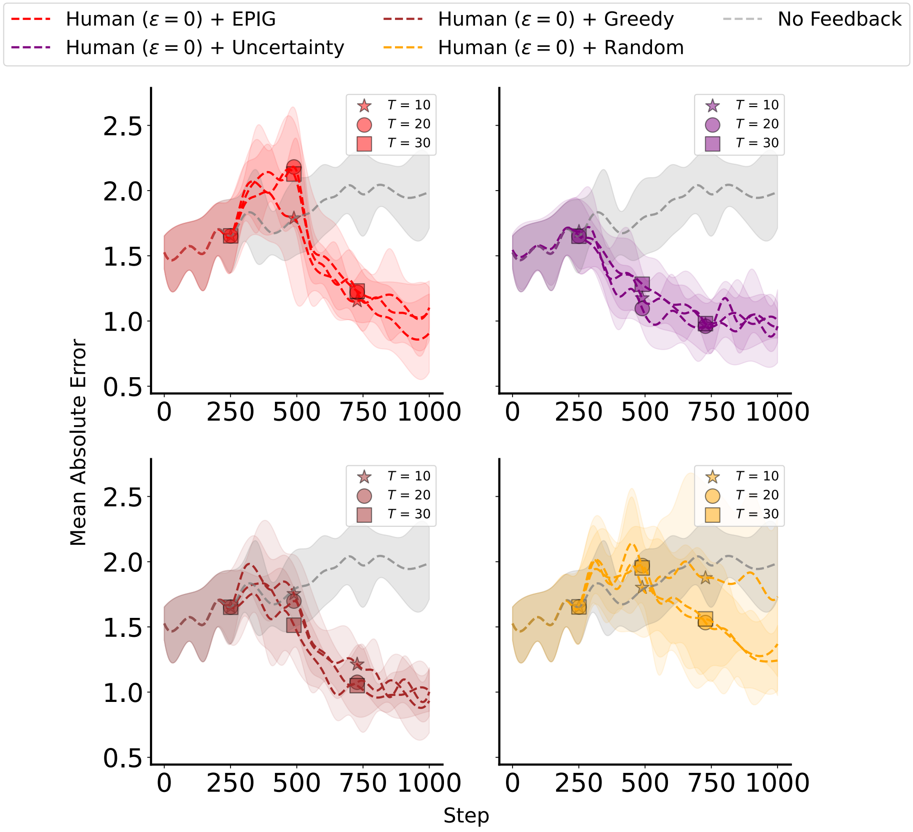

Figure S4: **Impact of the number of human queries for the penalized LogP optimization use case.** We report the mean and standard deviations across 10 replicates of each experimental run. For all acquisition criteria, we use a noise-free simulated expert queried every 250 steps of molecular generator optimization.

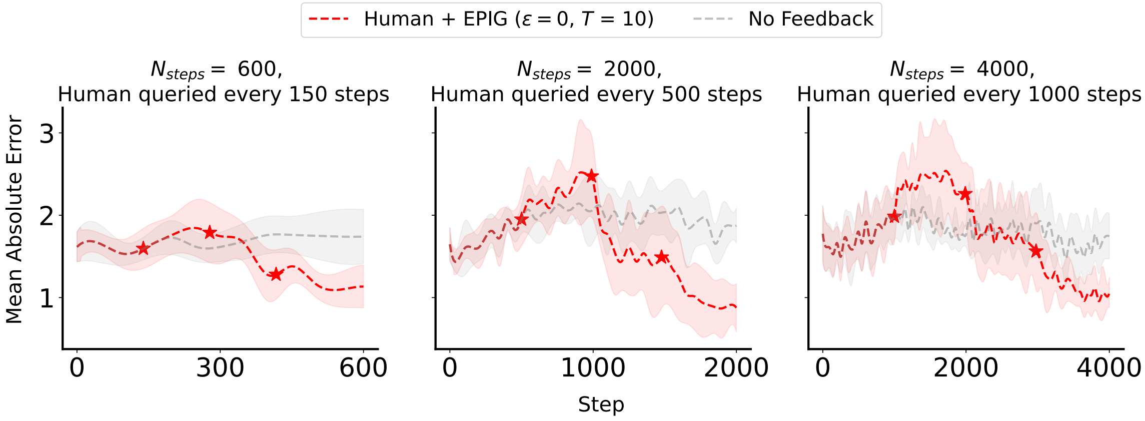

Figure S5: **Impact of the frequency of human queries for the penalized LogP optimization use case.** We report the mean and standard deviations across 10 replicates of each experimental run. For all acquisition criteria, we use a noise-free simulated expert and a query budget $T = 10$.

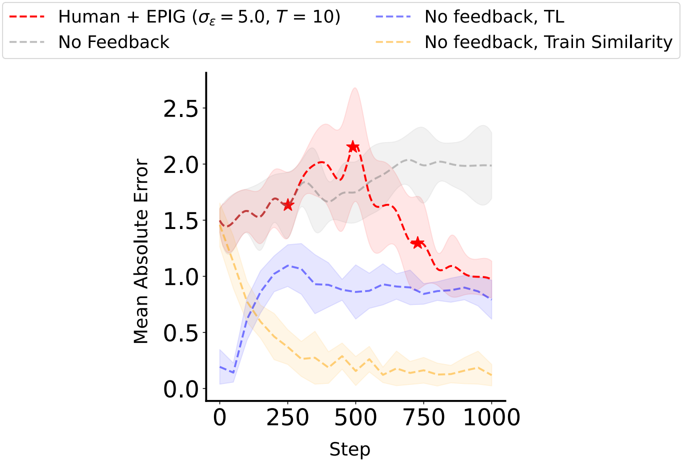

Figure S6: **Comparison with non-AL baselines for the penalized LogP optimization use case.** We report the mean and standard deviations across 10 replicates of each experimental run. We use a noise-free simulated expert queried every 250 steps of molecular generator optimization using EPIG.

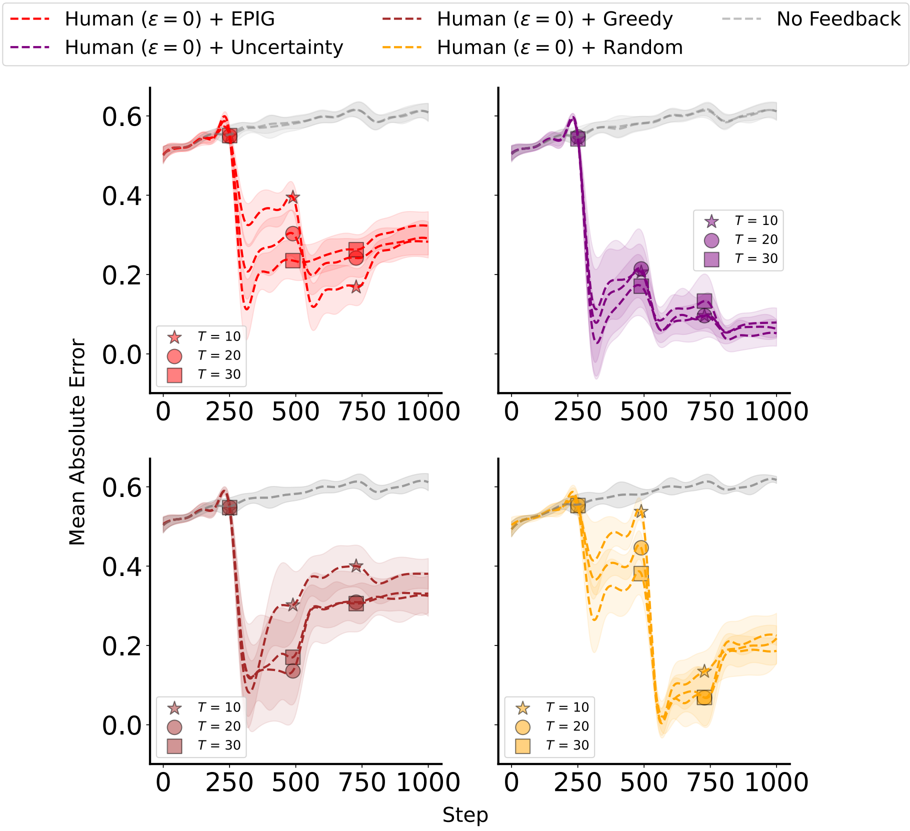

Figure S7: **Impact of the number of human queries for the DRD2 activity optimization use case.** We report the mean and standard deviations across 10 replicates of each experimental run. For all acquisition criteria, we use a noise-free simulated expert queried every 250 steps of molecular generator optimization.

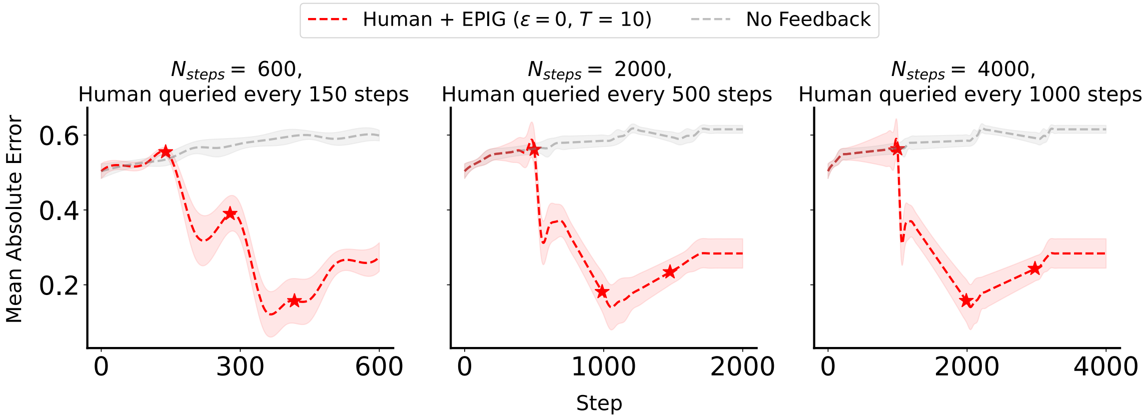

Figure S8: **Impact of the frequency of human queries for the DRD2 activity optimization use case.** We report the mean and standard deviations across 10 replicates of each experimental run. For all acquisition criteria, we use a noise-free simulated expert and a query budget $T = 10$.

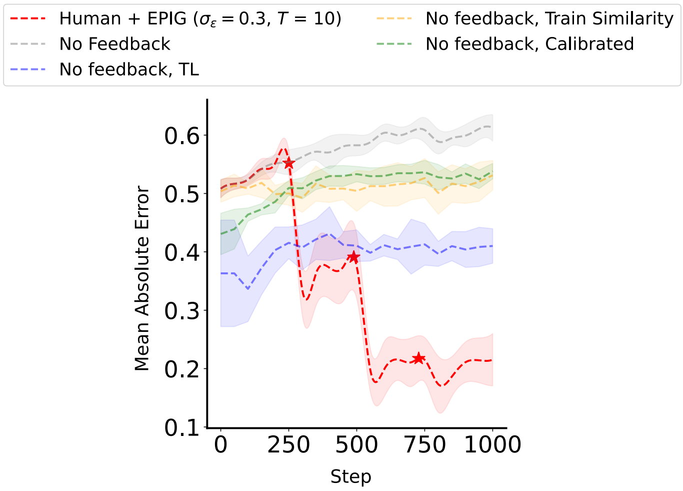

Figure S9: **Comparison with non-AL baselines for the DRD2 activity optimization use case.** We report the mean and standard deviations across 10 replicates of each experimental run. We use a noise-free simulated expert queried every 250 steps of molecular generator optimization using EPIG.

Additional results for the human experiments

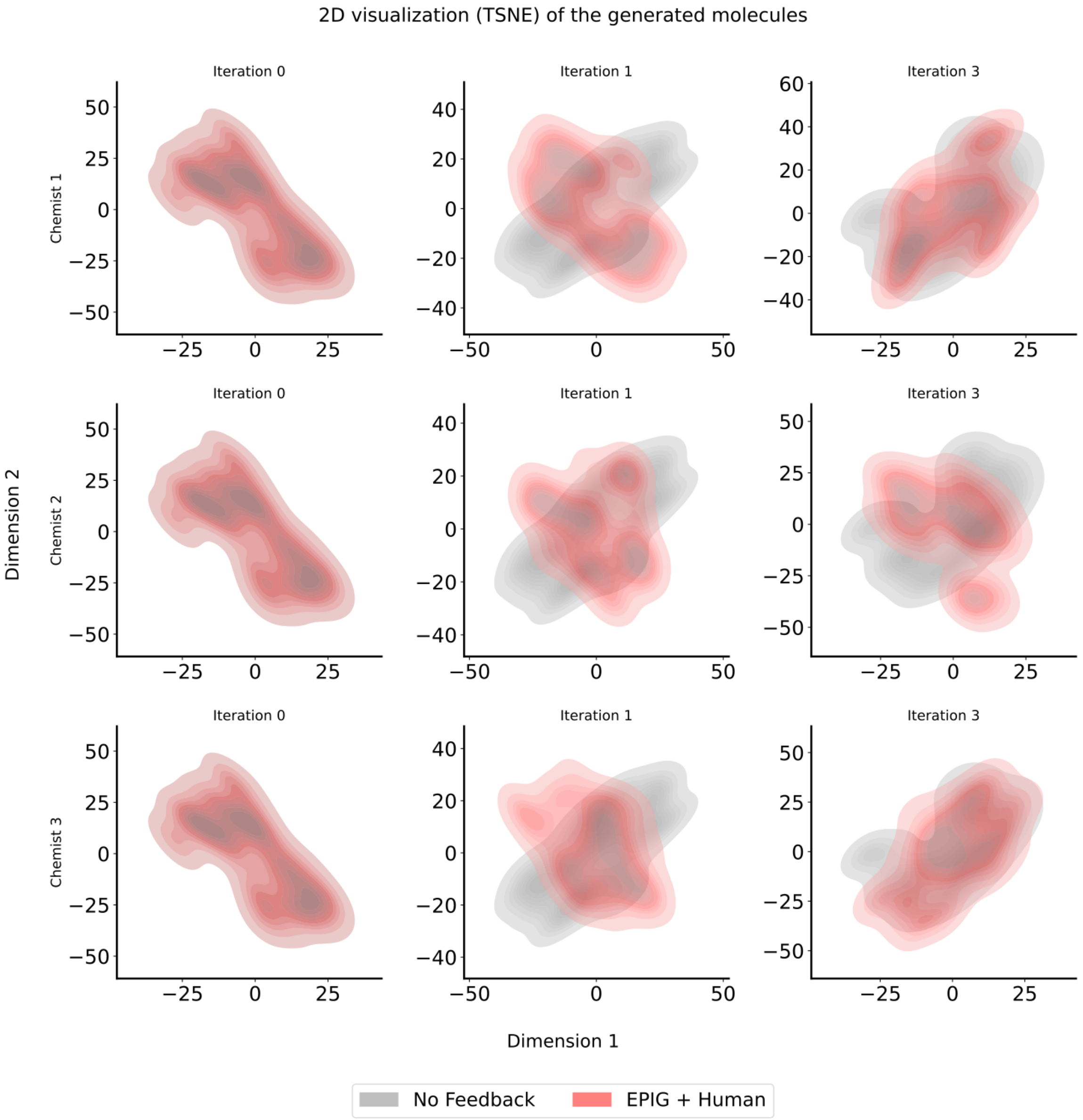

Figure S10: Distribution of high-scoring molecules generated during a multi-objective molecule generation with (in gray) and without (in red) intervention of chemist experts.
